# VirD4 coupling proteins suppress accumulation of conjugative pili produced by type IV secretion systems

**DOI:** 10.1128/mbio.01368-26

**Published:** 2026-07-16

**Authors:** Pratick Khara, Erin Hancock, Kouhei Kishida, Yang Grace Li, Peter J. Christie

**Affiliations:** 1 Department of Microbiology and Molecular Genetics, McGovern Medical School, UTHealth at Houston 12339 , Houston, Texas, USA; University of California, Berkeley, Berkeley, California, USA

**Keywords:** conjugation, horizontal DNA transfer, mobile genetic elements, type IV secretion, conjugative pilus, fluorescence microscopy

## Abstract

Conjugation machines constitute a large subfamily of the bacterial type IV secretion systems (T4SSs). In the absence of recipient cell contact, conjugation machines of gram-negative bacteria produce conjugative pili by extracting pilin subunits from an inner membrane (IM) pool. Upon pilus-mediated or direct contact with a target cell, conjugation systems transition to the recruitment of a cognate mobile element for intercellular DNA transfer. T4SS subunit requirements for pilus assembly and substrate transfer are nearly the same, except that VirD4 is required only for the latter. VirD4 ATPases (also termed type IV coupling proteins [T4CPs]) stimulate early DNA processing reactions and physically couple the DNA substrate to the T4SS channel. By visualizing fluorescently labeled pili, we identified marked differences in numbers, gross morphologies, and cellular organization of pili encoded by IncF F and IncN pKM101 plasmids. Remarkably, deletion of T4CP genes from both plasmids conferred hyperpiliation, a phenotype suppressed by complementation with the wild-type gene, but not alleles encoding variants defective in nucleotide binding/hydrolysis, or DNA substrate binding. In the pKM101 system, the T4CP plus TraK, a DNA processing factor that directs relaxosome assembly at the plasmid’s origin-of-transfer (*oriT*) sequence, also fully suppressed hyperpiliation even in the absence of *oriT*. We propose that the binding of a cognate DNA substrate (or certain relaxosome components) triggers the T4CP to suppress pilus production by blocking elongation, or stimulating retraction or release, which, in turn, transitions the T4SS from its default pilus-generating (mate-seeking) mode to the activated DNA-translocation (mating) machine.

**IMPORTANCE:**

Mobile genetic elements (MGEs), often with cargoes of antibiotic resistance and virulence determinants, disseminate widely among bacteria through their encoded type IV secretion systems (T4SSs). Newly assembled T4SSs produce conjugative pili, which promote attachment to biotic and abiotic surfaces. Upon pilus-mediated or direct contact with a recipient cell, T4SSs recruit type IV coupling proteins (T4CPs), which, in turn, promote MGE transfer through the T4SS channel. Through visualization of conjugative pili by fluorescence microscopy, we demonstrate that pili encoded by two model plasmids, IncF F and IncN pKM101, display gross differences in morphologies and spatial organization on *Escherichia coli* cells, implying the evolution of distinct strategies for adherence and mating pair formation. We further report that engagement of T4CPs and bound MGE substrates with the T4SS blocks pilus accumulation. We propose that the T4CP plays a critical role in transitioning the T4SS from its pilus production to DNA transfer modes.

## INTRODUCTION

Bacterial conjugation systems are medically problematic for their roles in dissemination of mobile genetic elements (MGEs) and their cargoes of antibiotic resistance and virulence determinants (1–3). Conjugation systems comprise one of the two main subgroups of type IV secretion systems (T4SSs), the second being the “effector translocators” functioning to deliver effector proteins to eukaryotic cells during infection (4, 5). In gram-negative bacteria, the T4SSs assemble from a minimum of 12 subunits generically termed VirB1–VirB11 and VirD4 (6). For the conjugation systems, the VirB components build an envelope-spanning nanomachine that, in the absence of contact-dependent signals from recipient cells, assembles an extracellular filament termed a conjugative pilus (7). In this mode, the T4SS recruits a specific type of substrate, pilin subunits embedded in the inner membrane (IM), to build the pilus. Upon pilus-mediated or direct donor-recipient cell contact, the T4SS transitions to recruit and translocate DNA substrates to the recipient cell (8, 9). Presently, signals governing the T4SS substrate switch from recruitment of IM-integrated pilins to cytoplasmic DNA cargoes are unknown.

Genetic requirements for production of the conjugative pilus and translocation channel are nearly the same, with the exception that VirD4 is needed only for substrate transfer (10, 11). Conjugation initiates when a relaxase and one or more accessory factors, termed mobilization (Mob) proteins, bind the origin-of-transfer (*oriT*) sequence of a mobile element. The resulting relaxosome nicks the DNA strand destined for transfer (T-strand), a reaction stimulated by binding of VirD4 (3, 6, 12). VirD4 also binds the T4SS channel, thus coupling the DNA substrate with the T4SS channel (6, 13–17). Upon binding the T4SS, substrate-docked VirD4 induces a conformational change in the distal portion of the channel required for the DNA substrate to pass into target cells (18–20). VirD4 subunits, also termed substrate receptors or type IV coupling proteins (T4CPs), thus play multiple, critical roles in stimulating DNA substrate processing, coupling the processed substrate to the T4SS, and activating the T4SS for DNA transfer.

In this study, we demonstrate that VirD4-like TraD and TraJ, respectively, encoded by the IncF F and IncN pKM101 conjugative plasmids, also regulate accumulation of conjugative pili. Because original studies of these systems adopted distinct nomenclatures for machine components, to clarify this presentation, we will append the subscripts F and pKM to the associated genes, proteins, and T4SSs, e.g., *traD_F_*, TraD_F_, and T4SS_F_. To monitor pilus production, we capitalized on a recent advance enabling direct visualization of cysteine-derivatized conjugative pili by fluorescence microscopy (21–24). By labeling Cys-derivatized TraA_F_ pilins, we confirmed previous findings that F-carrying *Escherichia coli* cells elaborate ~1–3 flexible F pili with lengths of ~1–2 μm (23, 25). In contrast, by fluorescent labeling the pKM101-encoded TraM_pKM_ pilins, we show here that pKM101-carrying cells elaborate abundant short N pili appearing as punctate fluorescent foci and only rarely produce detectable N pili extending from the cell surface. Remarkably, in both systems, deletion of the T4CP gene conferred hyperpiliation phenotypes that were reversed by expression of the wild-type (WT) genes but not by alleles with mutations known to disrupt T4CP–T4SS interactions, bind/hydrolyze ATP, and bind Mob DNA processing proteins. Together, our findings support a new model in which the engagement of the substrate-bound T4CP with the T4SS blocks further pilus elongation or, additionally or alternatively, stimulates pilus loss by promoting retraction, depolymerization, or release from cells. Ultimately, engagement of the substrate-bound T4CP triggers a transition in the T4SS from pilus production (mate-seeking mode) to channel activation and DNA transfer (mating mode).

## RESULTS

### pOX38-encoded TraD blocks F pilus production

**Fig 1:**
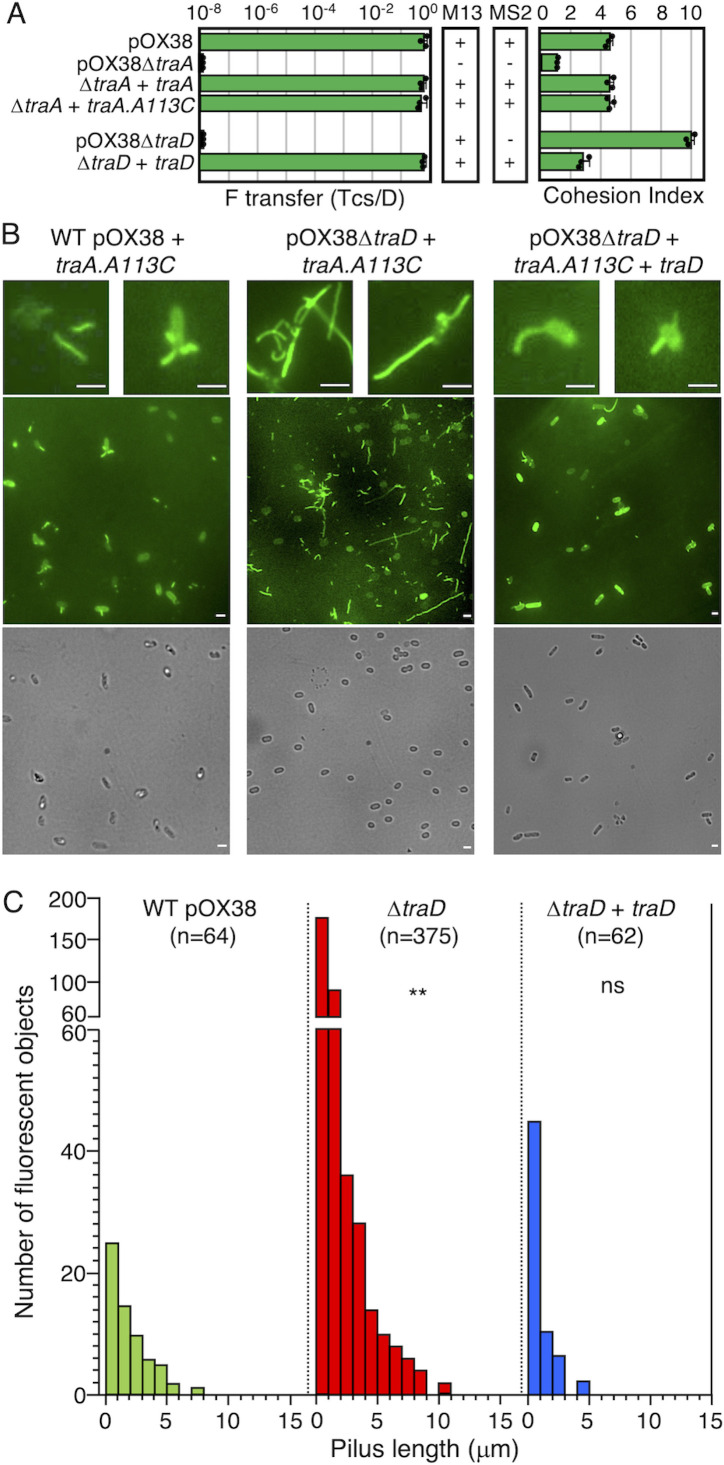
Deletion of the TraD_F_ T4CP confers elevated production of the F pilus. (**A**) Effects of TraA_F_ Cys-derivatization and the pOX38Δ*traD* mutation on F plasmid transfer (left), phage sensitivity (middle), and F pilus-mediated aggregation (right). Transfer frequencies of strains harboring pOX38, pOX38Δ*traA*, or pOX38Δ*traD,* with or without *trans* expression of the indicated alleles, are presented as the number of transconjugants/donor (Tc’s/D). The same strains were assayed for sensitivity to the male-specific phages M13 and MS2 as assessed by plaque formation (+ or −) in spot assays, and for pilus-mediated aggregation in a cohesion assay with the plasmid-free parental strain MC4100 as reference (set to 1). Matings and cohesion assays were repeated at least three times in triplicate; a representative experiment is shown, with replicate data points and average transfer frequencies as bars, along with standard deviations as error bars. (**B**) Fluorescence of AF488-mal-treated pili. Strains: MC4100 harboring the pOX38 variants shown expressing *traA.A113C* from pKKF082; the pOX38Δ*traD* strain lacked or carried pPK31 for expression of *traD_F_*. Top: magnified images of cells with attached F pili detected by fluorescent labeling using a FITC filter. Middle: representative fields of view for each strain, showing fluorescent pili/fragments attached to or detached from cells. Bottom: corresponding fields of view imaged with differential interference contrast (DIC). Scale bars, 2 µm. (**C**) Quantification of fluorescent objects representing intact F and pilus fragments. Objects identified by ImageJ in four representative fields of view were summed (*n* value) and numbers presented as a function of pilus/fragment length (in μm) determined by ImageJ for each strain. Background fluorescence of dim or aberrantly brightly fluorescing cells was subtracted prior to final quantifications. Not significant (ns), *P* > 0.05; **P < 0.01.

We introduced Cys residues near the C-terminus of the pilin subunit TraA_F_ encoded by the well-characterized IncFII plasmid pOX38 (26). One mutant protein, TraA.A113C_F_, was predicted by modeling with the published F pilus structure (27) to localize at the surface of the assembled F pilus (Fig. S1) (28). The *traA.A113C_F_* allele fully complemented a Δ*traA_F_* mutation in supporting transfer of the F plasmid*,* infection by male-specific bacteriophages MS2 and M13, and F pilus-mediated cellular aggregation (Fig. 1A). We detected Cys-derivatized F pili by FM488-mal labeling and fluorescence microscopy and, reminiscent of previous findings (23), approximately half the *E. coli* pOX38-carrying (WT strain) and pOX38Δ*traA* mutant cell populations engineered to express *traA.A113C_F_* from a separate plasmid produced from 1 to 3 F pili of ~0.5–2 μm in length, only occasionally longer (Fig. 1B; Fig. S2A) (28). While most F pili were cell-associated, detached F pili or pilus fragments also accumulated in the milieu. Next, we tested whether the TraD_F_ T4CP contributes to F pilus production by engineering a strain carrying pOX38 deleted of *traD* (pOX38Δ*traD*) to express *traA.A113C_F_*. In striking contrast to the WT pOX38-carrying strain, the Δ*traD_F_* mutant strain produced abundant F pili; many pili were aberrantly long, and pili and pilus fragments were abundant in the milieu (Fig. 1B; Fig. S2B). The pOX38Δ*traD* mutant strain exhibited extensive aggregation, as monitored by a cohesion assay (Fig. 1A). Consistently, aggregates of cells and pili were commonly visualized by fluorescence microscopy (Fig. S2C). Indeed, the bright fluorescence emitted by cell aggregates impeded detection of isolated cells and pili in the same fields of view, rendering it necessary to extensively scan for dispersed cell populations (Fig. 1B; Fig. S2B). As expected, *traD_F_* expression from a separate plasmid restored pilus production and aggregation to WT levels (Fig. 1A and B; Fig. S2D). Overall, these findings agree with our prior evidence that the pOX38Δ*traD* mutant elaborates more and longer pili than WT pOX38-carrying cells (11) (see Materials and Methods).

To quantify pilus production, we grew strains to equivalent CFU’s and collected multiple fluorescent microscopy images for each strain containing similar numbers of dispersed cells. Using ImageJ, we summed the total number and lengths of fluorescent objects in four fields of view for each strain to ensure unbiased sampling. We removed background fluorescence of intact cells and, confirming our visual observations, the pOX38Δ*traD* mutant produced significantly more pili or pilus fragments than the WT strain (Fig. 1C). We also detected a broader range of pilus lengths, ranging in size from ~0.5 µm to >10 µm, the lower end likely representing short pili and fragments and the higher to aberrantly long, presumptively intact pili. Cell-associated F pili were clearly visible, although detached pili/fragments were also highly abundant. Quantifications confirmed that expression of *traD_F_* restored pilus production by the pOX38Δ*traD* mutant to WT levels (Fig. 1C). Together, our findings suggest that the TraD_F_ T4CP controls F pilus accumulation both quantitatively on a per cell basis and in length. We also note that, due to extensive aggregation of the pOX38Δ*traD* mutant, the quantification of images populated by dispersed cells and pili likely underestimates the actual differences in pilus production by WT pOX38-carrying cells vs the pOX38Δ*traD* mutant.

**Fig 2:**
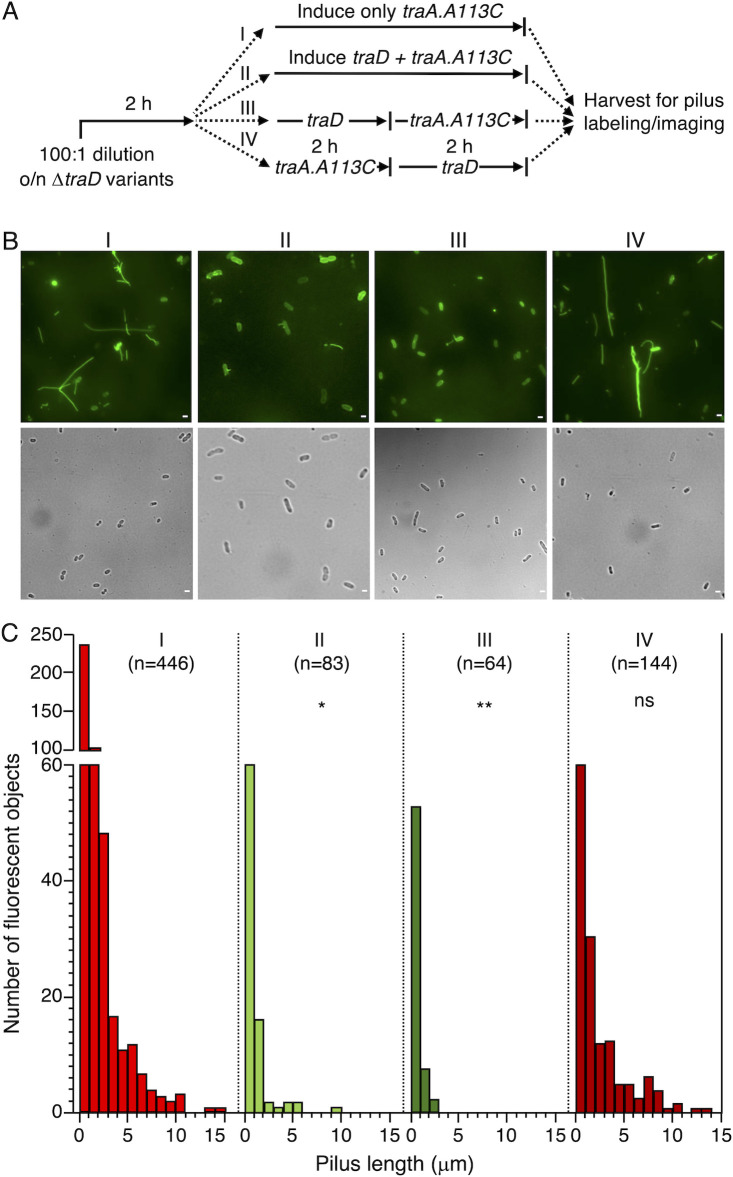
Temporal regulation of *traA_F_.A113C* vs *traD_F_* expression yields evidence for a TraD_F_-mediated block in F pilus production. (**A**) Schematic showing four variations (I–IV) in expression of *traA_F_.A113C* and/or *traD_F_* over the course of the 4-h induction period (see text for details). (**B**) Fluorescence of AF488-mal-treated cells and associated pili. Strains: MC4100 harboring pOX38Δ*traD* and plasmids with different tunable promoters for expression of *traA_F_.A113C* (pKKF082) and/or *traD_F_* (pPK31). Upper: representative fields of view for each strain, showing fluorescent pili/fragments attached to or detached from cells. Lower: corresponding fields of view imaged with DIC. Scale bars, 2 µm. (**C**) Quantification of fluorescent objects representing intact F and pilus fragments, as described in Fig. 1 legend. Numbers of fluorescent F pili and pilus fragments elaborated by the pOX38Δ*traD* induced for expression of *traA.A113C* (condition I) were compared with those elaborated under conditions II, III, and IV. Not significant (ns), *P* > 0.05; *P < 0.05; **P < 0.01.

We envisioned that TraD_F_ might dock early during biogenesis of the T4SS to block F pilus production entirely, or with a T4SS actively engaged in pilus production to halt further elongation or stimulate retraction. To gain evidence for one or both possibilities, we differentially controlled the timing of TraD_F_ synthesis and production of fluorescent F pili (Fig. 2A). To this end, we engineered the pOX38Δ*traD* mutant strain to express *traD_F_* and *traA.A113C_F_* from different, tunable promoters. In condition I, we induced expression only of *traA.A113C_F_* and, as expected, we observed hyperpiliation, supporting our proposal that T4SS defaults to pilus production in the absence of TraD_F_ (Fig. 2B and C). In condition II, we co-expressed *traD_F_* and *traA.A113C_F_* for 4 h and observed production of F pili in numbers and lengths comparable to a WT pOX38-carrying strain, a statistically significant reduction in overall pilus production compared with condition I (Fig. 2B and C). When TraD_F_ and TraA.A113C_F_ are co-produced, TraD_F_ clearly antagonizes pilus accumulation.

In conditions III and IV, we induced expression of *traD_F_* (III) or *traA.A113C_F_* (IV) for 2 h, then induced expression of the second gene for another 2 h (Fig. 2A). In condition III, with initial production of TraD_F_, we observed even stronger suppression of pilus accumulation than achieved when *traD_F_* and *traA.A113C_F_* were coexpressed throughout the entire 4-h period (Fig. 2B and C). These findings are most consistent with the idea that TraD_F_ binds available quiescent T4SSs produced during the first 2 h, effectively impeding the transition to pilus production. During the subsequent 2 h, co-produced TraD_F_ and TraA.A113C_F_ compete for the remaining or any newly synthesized T4SSs, yielding only a few pilus-generating machines. In condition IV, with initial production of TraA.A113C_F_, cells clearly produced more F pili than observed with conditions II and III, but fewer pili than with condition I, although the apparent reduction was not statistically significant (Fig. 2B and C). We posit that, during the first 2 h, most quiescent T4SSs transitioned to pilus-generating machines, resulting in the observed level of hyperpiliation. During the subsequent 2 h, co-produced TraD_F_ and TraA.A113C_F_ compete for any remaining available T4SSs, yielding a few additional pilus-generating machines. We confirmed that induction of *traD* expression in the pOX38Δ*traD* mutant strain for the times indicated yielded abundant amounts of TraD (Fig. S3A). Similarly, induction of *traA.A113C* yielded increased levels of TraA.A113C compared with amounts of native TraA pilin produced by the pOX38Δ*traD* mutant in the absence of induction (Fig. S3A).

### Catalytically active and substrate-binding-proficient forms of TraD_F_ are necessary to regulate F pilus production

**Fig 3:**
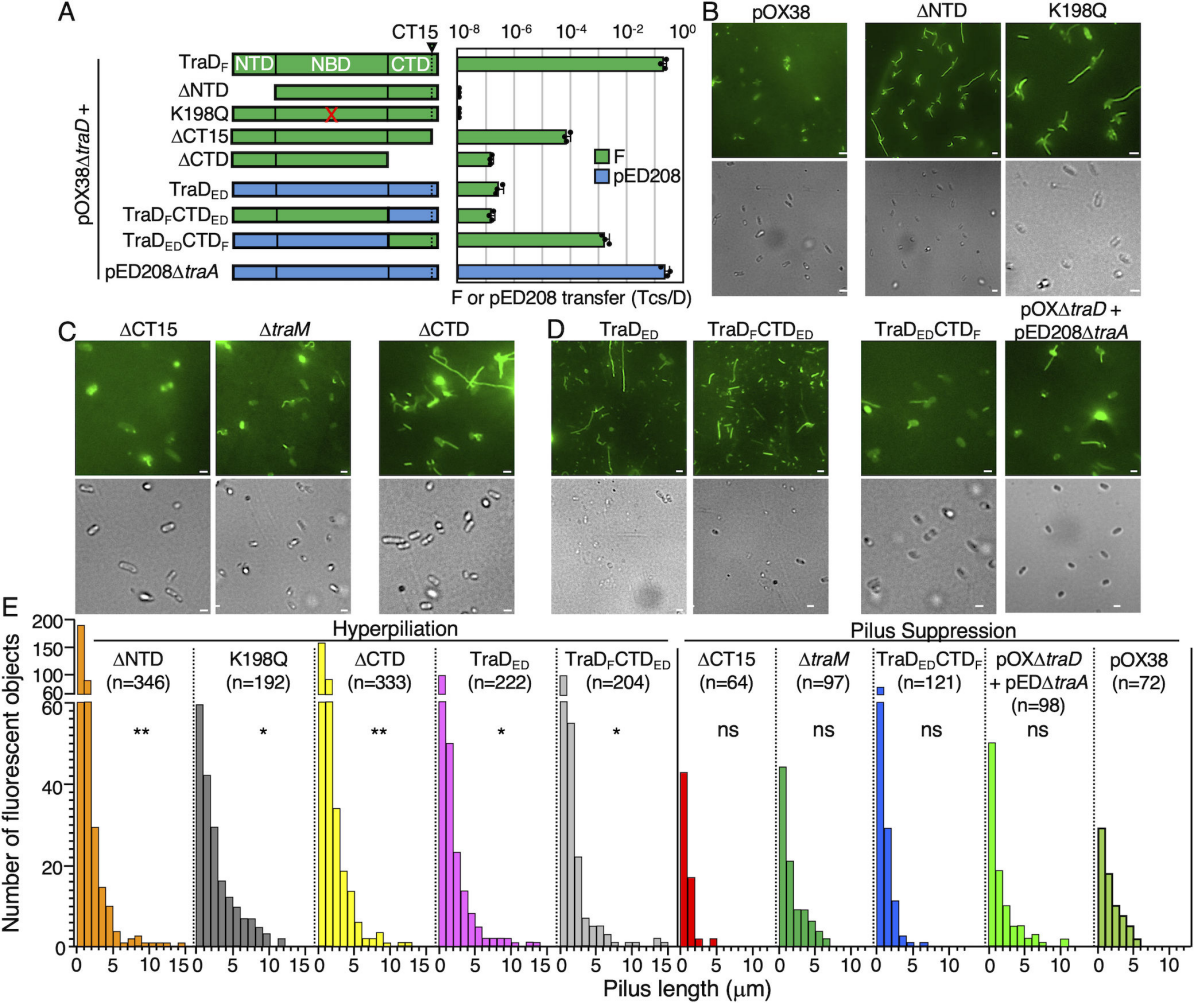
Engagement of the TraD_F_ T4CP with cognate plasmid processing factors is required for suppression of F pilus production. (**A**) Schematic showing TraD variants produced in the pOX38Δ*traD* host (left) and their functionality as assessed by 1.5-h broth mating assays (right). TraD_F_ domains are presented at the top: NTD, N-terminal transmembrane domain; NBD, nucleotide-binding domain; CTD, C-terminal domain responsible for plasmid substrate binding; CT15, C-terminal 15 residues bearing the TraM_F_-interaction motif. The TraD_F_ variants or TraD_F_/TraD_ED_ chimeras responsible for mediating plasmid transfer are represented by green (derived from pOX38-encoded TraD_F_) or blue (derived from pED208-encoded TraD_ED_) bars. Transfer frequencies (Tc’s/D) are presented for transmission of pOX38Δ*traD* (green) or pED208Δ*traA* (blue); matings were carried out in triplicate, and frequencies are presented for a representative mating. (**B–D**) Fluorescence of AF488-mal-treated F pili/fragments. Strains: MC4100 cells carrying pOX38 or pOX38Δ*traD* without or with plasmids engineered to produce the TraD variants depicted in panel **A**. Scale bars, 2 µm. (**E**) Quantification of fluorescent objects corresponding to intact F and pilus fragments, as described in the Fig. 1 legend. Not significant (ns), *P* > 0.05; *P < 0.05; **P < 0.01.

Next, we interrogated the TraD_F_ domain requirements for controlling F pilus production. T4CPs assemble as homohexamers with at least three distinct domains required for protein function (Fig. 3A) (17, 29, 30). The N-terminal transmembrane domain (NTD) interacts with inner membrane components of the T4SS to couple bound secretion substrates to the translocation channel (15, 31, 32). The nucleotide-binding domain (NBD) binds and hydrolyzes ATP to stimulate processing of DNA substrates and activate the translocation channel (29, 33–35). T4CPs additionally possess C-terminal domains (CTDs), as in the case of F-encoded TraD (22, 36, 37), or all-alpha domains (AADs), as in the case of pKM101-encoded TraJ (38–40) (see below), that mediate contacts with DNA or protein substrates (41).

We analyzed the impact of deleting or mutating TraD_F_’s NTD, NBD, and CTD on F pilus accumulation. The pOX38Δ*traD* mutant producing TraDΔNTD_F_ from a separate plasmid produced abundant levels of the mutant protein (Fig. S3B), but failed to conjugatively transfer the F plasmid (Fig. 3A) and was hyperpiliated (Fig. 3B and E). These findings are in line with previous evidence that NTD-mutated forms of T4CPs fail to dock productively with the T4SS (32, 42). Similarly, a Gln substitution for the invariant Lys198 residue within the Walker A nucleotide-binding pocket was stably produced (Fig. S3B) but blocked plasmid transfer (Fig. 3A) and conferred hyperpiliation (Fig. 3B and E), suggesting that TraD regulates pilus accumulation by a mechanism dependent on nucleotide binding or hydrolysis.

TraD_F_’s ~148-residue CTD is required for F transfer at high frequencies (Fig. 3A) (22, 36). This is achieved partly through a structurally defined interaction between the last eight residues of TraD_F_ with TraM_F_ (37). TraM_F_ is a ribbon-helix-helix (RHH) DNA-binding protein that binds F’s *oriT* sequence and promotes formation of the catalytically active relaxosomal complex through recruitment of the TraI_F_ relaxase to the *nic* site within *oriT* (43, 44). The overall network of interactions, between F’s *oriT* with TraM_F_, TraM_F_ with TraD_F_’s CTD, and TraD_F_’s NTD with the T4SS_F_, physically couples the F plasmid substrate with the translocation channel. Deletion of the TraM_F_-binding domain of TraD_F_ (ΔCT15) diminishes F plasmid transfer by 10^3–4^-fold (22, 37), whereas deletion of the entire 148-residue CTD (ΔCTD) further attenuates transfer by another 10^2^-fold (Fig. 3A). These findings have led to proposals that TraD_F_’s CTD contributes to recruitment of the F plasmid substrate not just through its TraM_F_ interaction, but additionally through unspecified interactions with one or more other plasmid-encoded relaxosomal components, e.g., TraI_F_ relaxase (45, 46).

We assessed the effects of the TraD_F_ ΔCT15 and ΔCTD mutations, as well as a Δ*traM_F_* mutation, on F pilus production (Fig. 3C and E). Interestingly, a strain producing TraDΔCT15_F_ accumulated F pili at levels comparable to the WT strain, suggesting that TraD_F_ regulates pilus accumulation independently of the structurally defined TraD_F_–TraM_F_ interaction. Supporting this proposal, a pOX38Δ*traM_F_* mutant strain also elaborated F pili at levels comparable to WT cells. In contrast, the pOX38Δ*traD* strain producing TraDΔCTD_F_ elaborated abundant amounts of aberrantly long F pili and pilus fragments, reminiscent of the pOX38Δ*traD* hyperpiliation phenotype. TraD_F_ thus requires its 148-residue CTD to regulate the accumulation of F pili.

Recently, we explored the role of TraD’s CTD in specifying transfer of cognate vs noncognate F-like plasmids, specifically, IncFII F (pOX38) and the distantly related IncFV plasmid pED208 (22). Characterization of deletion mutations and chimeric T4CPs not only established the essentiality of the CTDs of the respective TraD’s for efficient substrate transfer, but also showed that the CTDs support efficient trafficking only of the cognate F-like plasmid. TraD’s CTD thus functions as a substrate specificity determinant likely through binding of relaxosomal components encoded by the cognate plasmid. Here, we asked whether congruity between the CTD and plasmid substrate was also required for TraD-mediated F pilus suppression.

We first produced pED208-encoded TraD (hereafter TraD_ED_) in a strain carrying pOX38Δ*traD*. This strain supported transfer of the pOX38 plasmid, albeit at low levels (~10^−7^ transconjugants/donor; Tc’s/D), and was hyperpiliated (Fig. 3D and E). Next, we produced two TraD chimeras in a strain carrying pOX38Δ*traD*, one consisting of pOX38-encoded TraD_F_ bearing the CTD from TraD_ED_ (designated TraD_F_CTD_ED_) and the second of TraD_ED_ bearing the CTD from TraD_F_ (TraD_ED_CTD_F_) (Fig. 3A). The TraD_F_CTD_ED_-producing strain transferred the pOX38 plasmid at low levels (~10^−7^ Tc’s/D) and was hyperpiliated**,** reminiscent of the phenotypes accompanying production of TraD_ED_ in the pOX38Δ*traD* background (Fig. 3D and E). In contrast, the pOX38Δ*traD* mutant engineered to produce TraD_ED_CTD_F_ efficiently transferred pOX38 (10^−1^ Tc’s/D) (Fig. 3A) and produced F pili at levels comparable to the pOX38-carrying WT strain (Fig. 3D and E compare with Fig. 1B and C). Importantly, we previously reported that all of the TraD variants bearing CTD deletions or swaps accumulate at abundant levels (22), ruling out the possibility that the observed phenotypes result from instability of the mutant proteins.

In our previous study, we also showed that two donor strains, one harboring pOX38Δ*traD* and a second pED208Δ*traA,* fail to transfer the mutant plasmids due to the essentiality of the TraD T4CP and TraA pilin (22). However, these two F plasmids can stably coexist in the same strain, and such a strain efficiently transfers pED208Δ*traA* at a high frequency (10^−1^ Tc’s/D) and pOX38Δ*traD* at a low frequency (10^−7^ Tc’s/D). We further demonstrated that transfer of both co-resident plasmids results from assembly of a functional T4SS_F_/TraD_ED_ chimeric machine (22). Here, we introduced the *traA.A113C_F_* expression plasmid into the strain harboring both pOX38Δ*traD* and pED208Δ*traA* and observed that this strain generates pOX38-encoded F pili at WT levels (Fig. 3D and E). Thus, while the T4SS_F_/TraD_ED_ chimeric machine fails to regulate pilus accumulation when the host strain carries only pOX38, this same chimeric machine efficiently suppresses F piliation when the host strain additionally carries a pED208 plasmid substrate.

Taken together, our findings firmly establish that TraD’s CTD must efficiently engage with its cognate DNA substrate for high-frequency plasmid transfer and, concomitantly, suppression of F piliation.

### pKM101-carrying cells are abundantly decorated with fluorescent foci and short N pili

**Fig 4:**
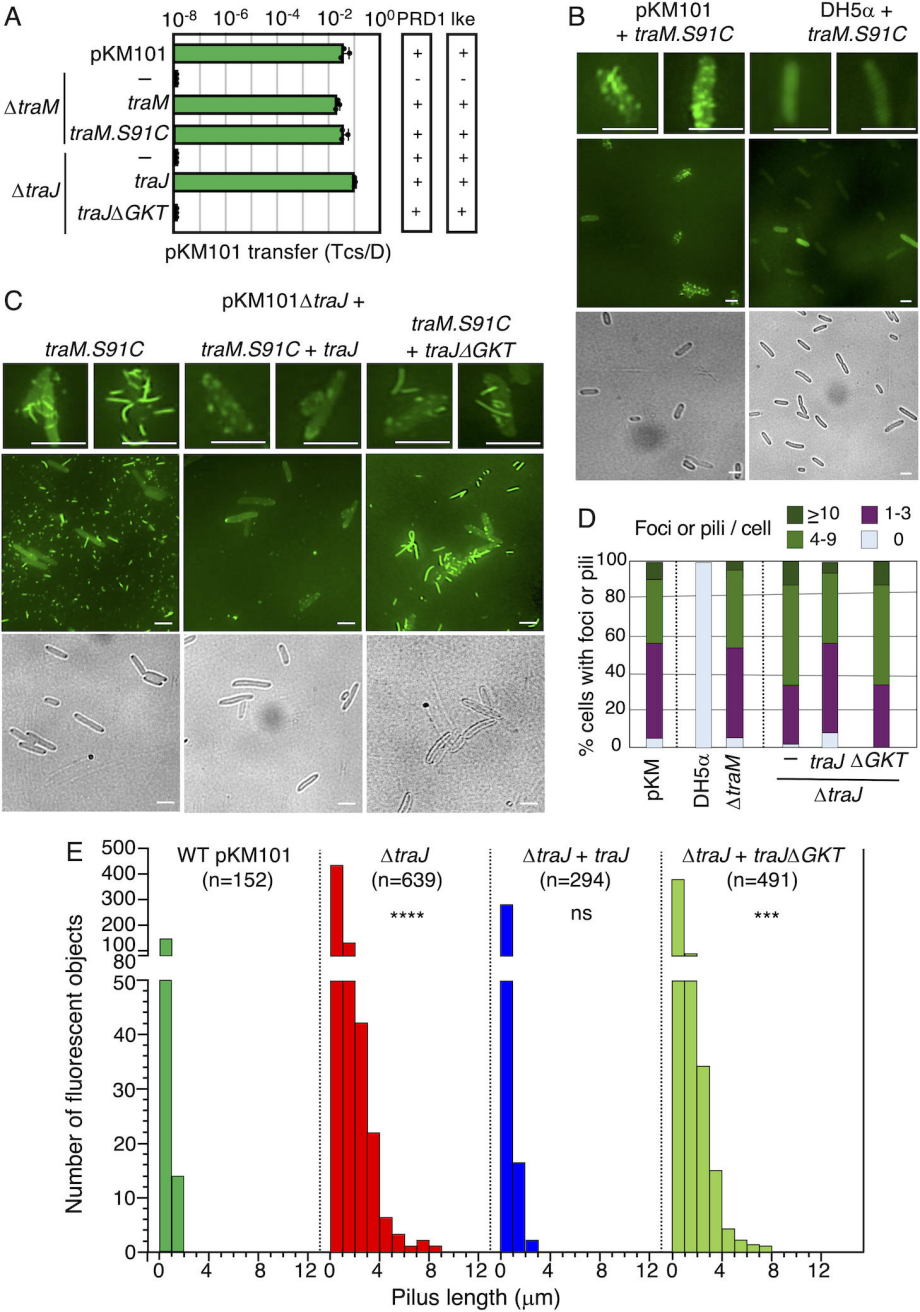
Deletion of the TraJ_pKM_ T4CP confers elevated production of the pKM101-encoded N pilus. (**A**) Effects of TraM_F_ Cys-derivatization and the pKM101Δ*traJ* mutation on pKM101 plasmid transfer (left) and phage sensitivity (right). Transfer frequencies of DH5α strains harboring pKM101, pKM101Δ*traM*, or pKM101Δ*traJ,* with or without *trans* expression of the indicated alleles, are presented as the number of transconjugants/donor (Tc’s/D). Matings were repeated at least three times in triplicate; a representative experiment is shown, with replicate data points and average transfer frequencies as bars, along with standard deviations as error bars. The same strains were assayed for sensitivity to the male-specific phages PRD1 and Ike as assessed by plaque formation (+ or −) in spot assays. (**B**) Fluorescence of AF488-mal-treated N foci and pili. Strains: DH5α lacking or carrying pKM101 plus pKKF090, which expresses *traM_pKM_S91C*. Top: magnified images of cells with attached N pili detected by fluorescent labeling using a FITC filter. Middle: representative fields of view for each strain, showing fluorescent pili/fragments attached to or detached from cells. Bottom: corresponding fields of view imaged with DIC. (**C**) Fluorescence of N foci/pili produced by strains carrying pKM101Δ*traJ* and pKKF090, either lacking or expressing *traJ_pKM_* or *traJ_pKM_*Δ*GKT*. Scale bars, 2 µm. (**D**) Numbers of fluorescent N foci or pili per cell, presented as a percentage of cells with the number of fluorescent objects indicated (color-coded; key at top) for a total of 50 cells analyzed per strain. DH5α strains carried pKM101 (pKM), pKM101Δ*traM* (Δ*traM*), or pKM101Δ*traJ* lacking or expressing *traJ_pKM_* or *traJ_pKM_*Δ*GKT*. All strains carried pKKF090 for expression of *traM_pKM_S91C*. (**E**) Quantification of fluorescent objects representing intact N pili and pilus fragments, as described in Fig. 1 legend. Not significant (ns), *P* > 0.05; **P < 0.01; ***P < 0.001; ****P < 0.0001.

Prior work has shown that F pili dynamically extend and retract, and that they preferentially localize at the midcell (23, 25). By contrast, Bradley’s early studies established that IncN or IncW plasmids encode more rigid pili that readily break from the cell (47, 48). We capitalized on our ability to visualize conjugative pili to characterize morphological and spatial features of IncN pKM101 plasmid-encoded N pili. We substituted Cys for residues near the termini of the pKM101-encoded TraM pilin, which are predicted to localize along the surface of the assembled N pilus (Fig. S1A) (49). Of seven substitution mutants tested, TraM.S91C_pKM_ exhibited wild-type function in fully supporting pKM101 plasmid transfer and infection by the male-specific phages Ike and PRD1 (Fig. 4A).

In striking contrast to the long, flexible F pili, pKM101-carrying cells producing TraM.S91C_pKM_ routinely possessed multiple fluorescent puncta or foci, and only a small subset of cells elaborated fluorescent N filaments projecting detectably from the cell surface (Fig. 4B). A strain carrying the pKM101Δ*traM* variant and producing TraM.S91C_pKM_ from a separate plasmid exhibited fluorescence patterns resembling those observed for the isogenic pKM101-carrying strain (Fig. S2E). We confirmed that the fluorescent foci and filaments corresponded to N pili by evaluating the labeling patterns of DH5α cells producing TraM.S91C_pKM_ in the absence of the pKM101-encoded T4SS (Fig. 4B), as well as plasmid-free DH5α cells and strains carrying pKM101 mutant plasmids deleted of genes encoding the VirB4-like TraB_pKM_ or VirB11-like TraG_pKM_ ATPases, which are essential for pilus production (Fig. S2H and I and J) (50). All strains exhibited only weak uniform fluorescence and no detectable pili or punctate foci. Among all control strains, we invariably detected cells with uniformly bright fluorescence or that possessed fluorescent puncta at one or both poles. We attribute these fluorescent features to nonspecific labeling of nonviable cells or cell poles.

We quantified the total number of fluorescent objects (punctate foci and N pili) per cell for 50 cells from imaged strains (Fig. 4D). As expected, strains defective for N pilus production lacked fluorescent foci/pili; only one or two cells exhibited background patterns of bright uniform or polar fluorescence. For strains competent for N pilus production, nearly all 50 imaged cells possessed foci/pili, with numbers ranging from 1 to >10. N pili or foci were distributed apparently randomly around the cell periphery, without preference for the midcell, as shown for F plasmid-encoded F pili (23).

Next, we quantified the total number of fluorescent objects associated with and detached from cells as a function of length for four fields of view per strain, following the protocol developed above for the F pilus (Fig. 4E). pKM101-carrying (WT) cells produced abundant numbers of fluorescent puncta of <1 µm in diameter, and comparatively few discernible N pili of 1–2 μm in length. These prominent features—abundant, uniformly distributed fluorescent foci and sparse numbers of short pili—readily distinguish the N pili elaborated by pKM101-carrying cells from the long and flexible F pili elaborated by F-carrying cells. We acknowledge that the observed patterns might be attributable to the reported brittle nature of N pili (47, 48) and that extensive fragmentation during sample preparation might yield only N puncta and short pili. However, we show below that various pKM101 mutant strains elaborate highly abundant, aberrantly long N pili, which clearly withstand extensive breakage during sample preparation. We therefore conclude that cells carrying WT pKM101 naturally elaborate abundant numbers of very short N pili appearing as puncta distributed around the cell peripheries, and rarely produce N pili longer than ~1 µm.

### pKM101-encoded TraJ inhibits N pilus production

Next, we compared N pilus production by pKM101-carrying cells and an isogenic strain deleted of *traJ_pKM_*, the gene encoding the TraJ_pKM_ T4CP (40, 50). Remarkably, the pKM101Δ*traJ* mutant strain elaborated N pili in significantly greater numbers and lengths than cells carrying the WT plasmid (Fig. 4C through E; Fig. S2F). Nearly all pKM101Δ*traJ* mutant cells had abundant fluorescent foci, and many also possessed aberrantly long N pili attached to the cell surface. Additionally, detached N pili or fragments were abundantly present in the milieu. Cell-associated or detached N pili ranged from 0.5 to 9 μm. As expected, complementation of the Δ*traJ_pKM_* mutation with *traJ* expressed in *trans* restored pilus abundance to levels and lengths observed for pKM101-carrying cells (Fig. 4C and E; Fig. S2G). In addition, a TraJ mutant protein deleted of the conserved Gly/Lys/Thr residues (ΔGKT) within the Walker A nucleotide-binding pocket of TraJ_pKM_ was stably produced in the pKM101Δ*traJ* host (Fig. S4A) but failed to support DNA transfer (Fig. S4B) or suppress N hyperpiliation (Fig. 4C through E). Thus, reminiscent of the F system, the pKM101-encoded T4CP requires an intact ATP-binding site to regulate N pilus abundance.

### The relaxosome processing factor TraK_pKM_ suffices for the TraJ_pKM_-mediated block in N piliation

**Fig 5:**
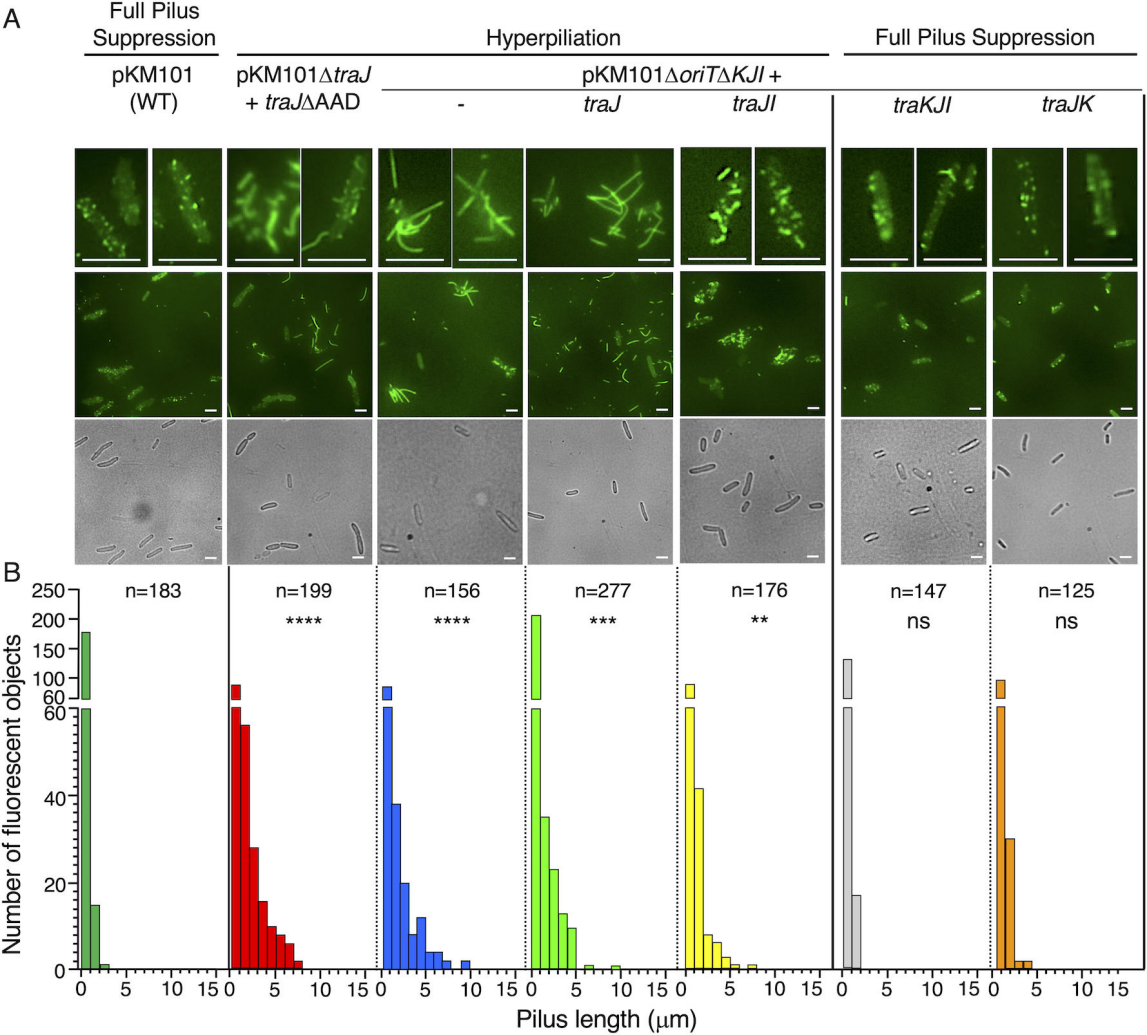
The TraK_pKM_ processing factor acts together with and independently of TraJ_pKM_ T4CP to suppress N pilus production. (**A**) N pili/fragments produced by strains carrying plasmids and expressing alleles listed at top visualized by AF488-mal labeling. All strains carried pKKF090 for expression of *traM_pKM_S91C*. Panels are as described in Fig. 4B. Strains exhibiting WT levels of N pili/fragments (pilus suppression) or pilus overproduction (hyperpiliation) are indicated. Scale bars, 2 µm. (**B**) Quantification of fluorescent objects corresponding to intact N pili and pilus fragments, as described in the Fig. 1 legend. Not significant (ns), *P* > 0.05; **P < 0.01; ***P < 0.001; ****P < 0.0001.

As demonstrated for other T4CPs (38, 39), TraJ_pKM_ is proposed to recruit the pKM101 substrate through interactions between its AAD and components of the relaxosome assembled at the plasmid’s *oriT* sequence. These factors include the TraI_pKM_ relaxase and TraK_pKM_, which, like F-encoded TraM (45), is a member of the RHH family of DNA-binding proteins (51). We deleted TraJ_pKM_’s AAD and determined that the TraJ_pKM_ΔAAD mutant protein was stably produced, but failed to support pKM101 transfer (Fig. S4A and B). This strain was also hyperpiliated (Fig. 5A and B), suggesting that TraJ_pKM_ requires its substrate-binding domain to regulate N pilus abundance.

Next, we interrogated the requirements for different relaxosome components for TraJ_pKM_-mediated suppression of N piliation (Fig. 5A and B). First, we deleted a contiguous region of pKM101 harboring *oriT*, *traK*, *traJ*, and *traI*, resulting in plasmid pKM101Δ*oriT*Δ*KJI*. As expected, pKM101Δ*oriT*Δ*KJI* was nontransmissible (Fig. S4B), and the strain carrying this plasmid was hyperpiliated (Fig. 5A and B). Interestingly, the pKM101Δ*oriT*Δ*KJI*-carrying host producing TraJ_pKM_ from a separate plasmid was also hyperpiliated (Fig. 5A and B). At the outset, we envisioned that TraJ_pKM_ must bind the relaxosome assembled at pKM101’s *oriT* sequence to regulate N pilus piliation. However, this was not the case, because the pKM101Δ*oriT*Δ*KJI*-carrying strain additionally producing TraK_pKM_, TraJ_pKM_, and TraI_pKM_ elaborated pili at levels comparable to cells carrying WT pKM101. As this strain lacks pKM101’s *oriT* sequence, TraJ_pKM_ must require only one or both plasmid processing factors, TraK_pKM_ and TraI_pKM_, but not their assembly as the catalytically active relaxosome at *oriT_pKM_*, to regulate N pilus levels.

These findings led us to ask whether TraJ_pKM_ requires only one of these processing factors to regulate N piliation. The pKM101Δ*oriT*Δ*KJI*-carrying strain producing only TraJ_pKM_ and TraI_pKM_ appeared slightly less hyperpiliated than the isogenic pKM101Δ*oriT*Δ*KJI* host, yet elaborated significantly more pili than the pKM101-carrying host (Fig. 5A and B). TraI_pKM_ thus does not markedly control pilus production through TraJ_pKM_. Remarkably, however, the pKM101Δ,*oriT*Δ*KJI*-carrying strain producing only TraJ_pKM_ and TraK_pKM_ was strongly suppressed for N piliation, with numbers and lengths comparable to pili elaborated by the WT pKM101-carrying strain (Fig. 5A and B). TraJ_pKM_ thus requires only the accessory factor TraK_pKM_ to fully suppress N piliation to levels observed with the pKM101-carrying WT strain.

## DISCUSSION

We capitalized on the ability to label Cys-derivatized conjugative pili with maleimide-fluorophore conjugates to compare spatial features of F and N conjugative pili. In contrast to the F system, which elaborates 1–3 long retractile F pili per cell, pKM101-carrying cells produce multiple fluorescent foci presumptively corresponding to very short pili, rarely producing N pili extending detectably from the cell surface. These differences in *in situ* cellular morphologies and spatial features lend further support to previous proposals, based on the flexible vs brittle nature of F and N pili, respectively (47), that F and pKM101 evolved different strategies for propagation. While long F pili endow donors with the capacity to transfer efficiently in both dense cellular and more dilute environmental settings, the short N pili favor efficient transfer only in dense conditions, e.g., polymicrobial biofilms. It is interesting to note that, although pKM101 does transfer at a ~10- to 100-fold lower frequency in liquid vs solid-surface conditions, transfer is still fairly robust, achieving frequencies of 10^−2^ Tc’s/D in 2-h liquid matings (50, 51). We previously showed that this is attributable to the additional elaboration of surface adhesins composed of two pKM101-encoded proteins, VirB5-like TraC and a protein termed Pep (52). TraC_pKM_ is required for N pilus assembly (50) and likely localizes at the tip of N pili (53), but both TraC_pKM_ and Pep_pKM_ are also exported to the cell surface independently of the T4SS_pKM_, where they interact to form higher-order adhesin complexes that promote intercellular contacts independently of N pilus production (52). pKM101 thus evidently evolved a dual strategy of abundantly decorating donor cells with short N pili as well as TraC/Pep_pKM_ surface adhesins to promote its transfer. It is also noteworthy that donor cells release N pili and the higher-order TraC/Pep_pKM_ complexes into the milieu (this study; 47, 52, 54, 55); these released adhesins can further amplify cellular aggregation and the probability of productive encounters in polymicrobial settings.

The short N pili generally resemble the conjugative pili recently visualized on the surfaces of *E. coli* cells carrying IncW plasmid R388 (24). Interestingly, in the R388 system, only 0.08% of plasmid-carrying cells elaborated a single, short W pilus. These cells were also devoid of distinct foci resembling the prevalent N foci of pKM101-carrying cells. Furthermore, significantly more R388-carrying cells (~14%) produced a W pilus when grown in the presence of plasmid-free recipient cells (24). We failed to observe enhanced production of N foci or pili in similar mating conditions, but since most pKM101-carrying cells in a donor population already elaborate multiple N foci/pili, such a recipient stimulatory effect might be difficult to quantify. The differences in numbers and spatial organization of N foci/pili vs W pili are of interest in view of structural findings supporting the classification of both the pKM101- and R388-encoded pilin subunits into a common family, which is distinct from the F plasmid-encoded TraA pilin family (24). N and W pilus filaments also share structural features, such as a common 5-start helical assembly and a 1:1 stoichiometric incorporation of phosphatidylglycerol (PG) per pilin (24, 49). There are, however, some structural differences between N and W pili, such as the nature of specific pilin-pilin and pilin-phospholipid contacts, as well as a splayed conformation of the W pilus-associated PG 32:1 that was not detected with N pilus-associated PG 34:1 (24). Whether these structural differences confer distinct physical properties that could account for the observed differences in N and W pilus abundance or spatial organizations, or the observed prevalence of N pilus fragments in culture supernatants, awaits further investigation.

There is ample evidence that T4CPs couple early-stage DNA substrate processing with delivery through the activated T4SS channel. The functions ascribed thus far include (i) T4CP interactions with Mob processing factors (37, 51, 56, 57), (ii) T4CP-mediated stimulation of relaxase nicking at the *nic* site within *oriT* to initiate DNA substrate processing (16), (iii) T4CP interactions with T4SS channel components (15, 31, 58), (iv) coordination of T4CP activities with the VirB ATPases to drive transfer of the processed DNA substrate through the T4SS channel (19, 31, 58), and (v) a T4CP-induced structural change in the T4SS channel of importance for DNA transfer through the distal portion of the channel (18, 19). Understanding how T4CPs orchestrate their functions at a structural level has been challenging, however, because to date, no high-resolution structures have been solved for relaxosome—T4CP or T4CP—T4SS channel interactions. T4CPs are members of the SpoIIIE/FtsK/HerA superfamily of dsDNA motor proteins and, accordingly, are envisioned to assemble as homohexameric DNA pumps at the entrances of T4SS channels (29). Although purified forms of T4CPs have been shown to assemble as homohexamers (29, 59–61), curiously, only one high-resolution study has thus far detected a T4CP in contact with the T4SS channel and, in this case, the T4CP (TrwB_R388_) assembled as two distinct dimers with the T4SS (62). The inability to detect homohexameric T4CP–channel complexes, coupled with other biochemical and cellular localization findings, has led to proposals that T4CPs associate only transiently or weakly with T4SSs and, upon engagement, both the T4CP and the T4SS undergo profound structural changes or oligomeric states (12, 62, 63).

Adding to these functional roles of T4CPs, we further determined that T4CPs associated with two model conjugation systems suppress the accumulation of conjugative pili. In fact, the notion that T4CPs somehow regulate pilus production surfaced decades ago with the observation that an amber mutation in *traD_F_* (designated *traD8*) affects the number of pili per cell (64). However, this early study did not evaluate whether a *traD8-*encoded fragment of TraD_F_ might artifactually impact F piliation, nor did this or subsequent work report quantitative effects of this mutation on pilus lengths or numbers. Here, we determined that the clean deletions of two T4CP genes, *traD_F_* and *traJ_pKM_*, significantly increase the numbers and lengths of F and N pili, and that these phenotypes are reversed by *trans*-complementation with the wild-type genes. We further supplied evidence that TraD_F_ and TraJ_pKM_ must also engage with the T4SS and bind or hydrolyze ATP, both expected outcomes based on the established importance of these activities for other T4CP functions. We note that our parallel studies of two distinct conjugation systems, with cross-system consistency in the outcomes, add rigor to our analyses and strengthen a general conclusion that T4CPs regulate the accumulation of conjugative pili through an ATP-binding-dependent mechanism.

Strikingly, we also determined that both T4CPs must carry intact DNA substrate-binding domains (TraD_F_ CTD, TraJ_pKM_ AAD) to suppress piliation, further suggesting that T4CPs act through a mechanism(s) that requires engagement with cognate DNA substrates. The importance of T4CP–substrate engagement was reinforced through our findings that TraD/CTD chimeras fully suppress F piliation only when the associated CTD is paired with a cognate F plasmid substrate. Interestingly, in the F system, the structurally defined TraD_F_–TraM_F_ interaction (37), which strongly contributes to but is not required for relaxosome assembly or F transfer (22, 43), does not detectably contribute to TraD_F_-mediated pilus suppression (Fig. 3). Given the essentiality of the entire CTD for WT plasmid transfer and pilus suppression, it is highly plausible that TraD_F_’s CTD must instead bind one or more other components of the relaxosome (TraI_F_, TraY_F_, *oriT*) to block piliation.

In the pKM101 system, we unexpectedly discovered that co-production of TraJ_pKM_ and TraK_pKM_, even in the absence of the TraI_pKM_ relaxase or the *oriT_pKM_* sequence, suffices to trigger TraJ_pKM_-mediated N pilus suppression. While this establishes that docking of the pKM101 substrate, e.g., the catalytically active relaxosome, is not required to activate TraJ_pKM_, we also note that TraK_pKM_ exhibits unusual functions not previously ascribed to other members of the RHH DNA-binding Mob factors. For example, we previously showed that TraK_pKM_ can activate the T4SS_pKM_ channel for DNA transfer independently of its role in promoting relaxosome assembly (51, 65). This activity requires binding of TraK_pKM_ to TraJ_pKM_, which, in turn, enables stable complex formation between TraJ_pKM_ and VirB4-like TraB_pKM_ ATPase (51). Formation of the TraK/TraJ/TraB_pKM_ ternary complex is a prerequisite for pKM101 transfer, as expected, but it is also required for transfer of the mobilizable plasmid RSF1010 through the T4SS_pKM_, even though RSF1010 encodes its own Mob processing proteins (51). Taken together with our present findings, the data suggest that TraK_pKM_-induced formation of the TraK/TraJ/TraB_pKM_ ternary complex acts simultaneously to block N piliation and activate the channel for DNA transfer. Although F-encoded TraM_F_ clearly does not carry out TraK_pKM_-like functions in promoting T4SS_F_ channel activation and F pilus suppression, it remains to be determined whether other members of the RHH Mob family, e.g., F-encoded TraY or RHH Mob factors associated with other conjugation systems, possess such attributes. For TraK_pKM_ itself, fascinating questions remain concerning its underlying mechanism(s) of action. Perhaps more fundamentally, why has a Mob protein that functions to process DNA substrates for transfer independently evolved the capacity to orchestrate T4CP-mediated pilus suppression?

**Fig 6:**
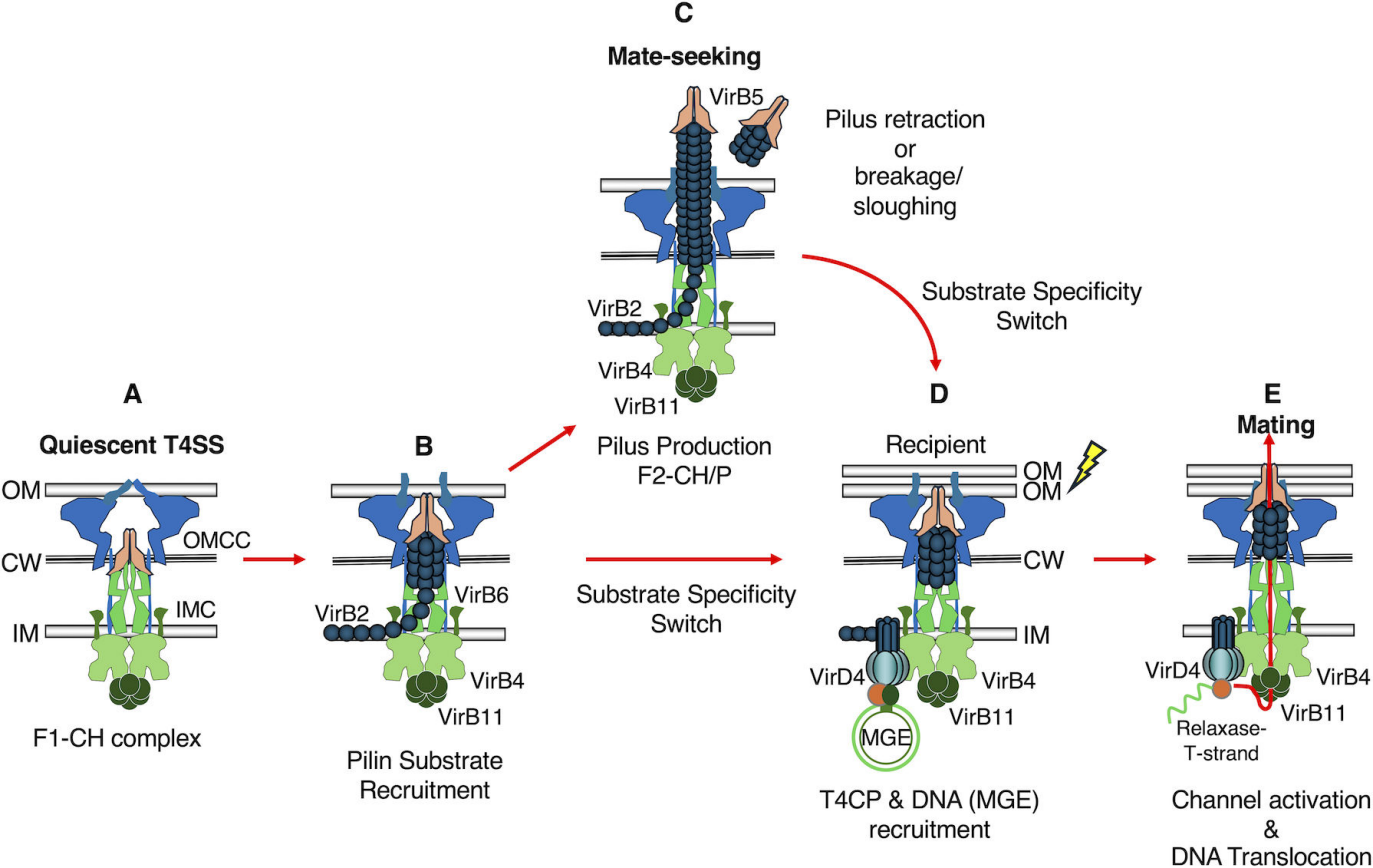
Model depicting the role of substrate-docked T4CP in regulating the pilin to DNA substrate specificity switch**.** (A) The T4SS assembles initially as a quiescent *trans*-envelope structure that (B, C) defaults to production of the conjugative pilus in a mate-seeking mode. (D) Upon docking of the substrate-engaged T4CP, the T4SS ceases recruitment of pilins from the inner membrane pool and undergoes conformational changes necessary for channel activation (opening). (E) Concomitantly, the T4CP coordinates with the VirB ATPases to orchestrate DNA substrate processing and delivery of the DNA transfer intermediate into the translocation channel in the mating mode. Abbreviations: F1-CH and F2-CH/P denote F-encoded quiescent and F-pilus-producing complexes, respectively, that we previously visualized by *in situ* CryoET; IM, inner membrane; OM, outer membrane; CW, cell wall; OMCC, outer membrane core complex; IMC, inner membrane complex; highly conserved VirB components include VirB11 hexameric ATPase, VirB4 hexamer-of-dimers ATPase, VirD4 T4CP ATPase, VirB2 pilin, and VirB6 and VirB5 pilus assembly/tip proteins; MGE, mobile genetic element; Relaxase-T-strand, DNA transfer intermediate.

Taken together, we propose an overarching model (see Fig. 6) depicting the late-stage engagement of the T4CP, along with its bound DNA substrate—or at least one or more components of the relaxosome—as a critical trigger for the T4SS to transition from its pilus-producing to mating modes. In line with our earlier structural findings for both the F- and pKM101-encoded transfer systems (66, 67), we propose that the T4SS initially assembles as a quiescent cell-envelope-spanning complex, which consists of all core structural subunits except for the VirB2-like pilin (Fig. 6A). In the F system, this corresponds to the quiescent structure that we previously visualized by *in situ* CryoET and designated as the F1-CH complex (66). Available data suggest that the pilin subunit is essential for DNA transfer, even among cells that lack detectable conjugative pili (68). Consequently, we propose that the quiescent machine begins to recruit pilin substrates from the IM pilin pool (Fig. 6B). The VirB4-like ATPase and, when present, the VirB11-like ATPase catalyze pilin dislocation from the IM, as evidenced by prior work in the *A. tumefaciens* VirB/VirD4 system (69). Furthermore, based on recent structural studies of the R388 conjugation machine (70), upon dislocation, each pilin subunit extracts a molecule of PG from the IM, and the resulting pilin::PG complexes then nucleate as the helical pilus fiber on a platform comprised of a VirB6-like subunit.

In the absence of recipient cell contact, many pilin::PG complexes are recruited to build the pilus fiber, which extends through the portion of the T4SS designated as the outer membrane core complex (OMCC) (Fig. 6C). The extruded pilus can dynamically extend and retract as shown for the F pilus (23, 25), extend only a short distance from the cell surface as shown here for N pili and elsewhere for W pili (24), or dislodge from the cell through breakage or an active sloughing mechanism as shown here for N pili. Upon receipt of a signal propagated from a recipient cell, the T4SS recruits the T4CP, which, in turn, recruits the DNA substrate (Fig. 6D; MGE). Although other roles are possible (see below), we favor a proposal that the T4CP/MGE complex impedes pilus elongation by blocking further recruitment of pilin:PG complexes. At a mechanistic level, the T4CP/MGE complex might block pilin dislocase activities of the VirB4(VirB11)-like ATPase(s), for example, by disrupting catalytic activities or inducing major conformational or oligomeric changes. In this latter context, it is intriguing to note that although the VirB4 ATPases have been shown to assemble as hexamers-of-dimers at the cytoplasmic entrances of several T4SSs (7, 66, 67, 71–73), this architecture has been visualized only among quiescent T4SS machines in the absence of bound T4CPs. Conceivably, T4CP/MGE docking triggers reorganization of the VirB4 ATPases, yielding alternative structures such as that visualized for the TrwB-docked TrwB T4SS, which consists of two TrwB dimers sandwiched between two hexameric barrels of VirB4-like TrwK (62). Such structural rearrangements at the base of the T4SS channel could function to drive substrate transfer while blocking pilin dislocation and pilus assembly.

If the T4CP/MGE complex is recruited to the T4SS early on, no detectable pili are produced (Fig. 6B through D). Although our temporal expression studies (Fig. 2) firmly established the capacity of the T4CP to block the T4SS from transitioning to its pilus production mode, in nature, we posit that the formation of direct donor–recipient contacts in dense growth conditions supplies the necessary signal(s) to trigger the observed T4CP/MGE-mediated block in piliation. If, on the other hand, the T4CP/MGE complex docks with a T4SS actively engaged in pilus production—which in the F system corresponds to a structure we visualized by *in situ* CryoET and designated as the F2-CH/P complex (66)—a T4CP/MGE-mediated block in a VirB4/VirB11 pilin dislocase function would terminate further pilus elongation (Fig. 6C through D). We acknowledge that the T4CP might act in different ways to suppress pilus lengths or numbers, for example, by stimulating pilus retraction or by triggering depolymerization or release of extended pili into the milieu. Of course, these different possible modes of action are not mutually exclusive; for example, the T4CP could act simultaneously to terminate pilus elongation and trigger retraction, both through modulation of VirB4’s pilin dislocase activity. Identifying the underlying function(s) of T4CPs in regulating piliation will require further time-resolved imaging to define contributions of T4CPs to pilus extension/retraction dynamics or pilus/pilin release.

Finally, regardless of whether the T4CP/MGE complex engages early on with the T4SS (Fig. 6B) or following rounds of pilus production (Fig. 6C), we propose that the T4CP induces a substrate specificity switch, from pilin:PG to DNA. Concomitantly, assembly of the T4SS/T4CP/MGE ternary complex stimulates processing of the MGE for transfer and activates the channel for DNA substrate passage (Fig. 6E). Although our model depicts DNA transfer between donor and recipient cells in direct contact, F-carrying cells also can translocate the F plasmid through the extended F pilus (23, 74, 75). It is plausible that transfer at a distance could occur if the T4CP/MGE docks with the pilus-generating complex, e.g., F2-CH/P, under specific conditions in which (i) the extended F pilus establishes contact with a recipient cell and (ii) the donor and recipient cells are physically constrained from being brought into apposition through F pilus retraction. Under such conditions, T4CP/MGE docking with the pilus-generating complex would collectively terminate further pilus elongation, stimulate DNA substrate processing, activate the channel, and drive substrate passage through the extended pilus.

## MATERIALS AND METHODS

### Bacterial growth conditions and plasmid constructions

*E. coli* strains listed in Table S1 were grown in Lysogeny Broth (LB) at 30°C for recombineering and at 37°C for other applications. For induction of *tra* genes expressed from inducible promoters, overnight-grown strains were first sub-cultured 1:50 in LB medium for 1.5 h and then induced with either arabinose (0.2% final concentration) for expression from the P_BAD_ promoter or sodium salicylate (1 µM final concentration) for expression from the P_nah_ promoter, and further grown for 1.5 h at 37°C. Strains and plasmids (Table S1) were maintained with appropriate antibiotics: spectinomycin (100 μg mL^−1^), carbenicillin (100 μg mL^−1^), kanamycin (100 μg mL^−1^), tetracycline (20 μg mL^−1^), chloramphenicol (20 μg mL^−1^), gentamicin (10 μg mL^−1^), and rifampicin (100 μg mL^−1^). All plasmids and oligonucleotides are listed in Tables S1 and S2, and details of plasmid constructions are presented in File. S1.

### Conjugation assay

Donor and recipient strains were grown overnight in LB medium supplemented with appropriate antibiotics. Strains were then diluted 1:50 in fresh antibiotic-free LB medium and incubated with shaking for 3 h at 37°C. Donor and recipient cultures were mixed in a 1:1 ratio and incubated at 37°C for 1.5 h without shaking. The mating mixtures were then serially diluted and plated onto LB agar containing antibiotics selective for transconjugants (Tc’s) and donors (D). The frequency of DNA transfer was calculated as (Tc’s/D). Matings were repeated at least three times in triplicate; a representative experiment is shown, with replicate data points and average transfer frequencies as bars, along with standard deviations as error bars.

### Phage sensitivity assay

Bacterial strains were cultivated as described above, and 80 µL of subcultured cells (induced or uninduced as required) were mixed with 5 mL of soft agar (0.75%) containing 4 mM CaCl_2_, which was then deposited onto an LB agar plate. When required, arabinose (0.2% final concentration) or sodium salicylate (1 µM final concentration) was also added to the soft agar. To assay the phage sensitivity of strains carrying pOX38 plasmids, 2 µL of MS2 (10^9^ pfu mL^−1^) or M13 (10^11^ pfu mL^−1^) phages were spotted on the solidified soft agar. To assay for phage sensitivity of strains carrying pKM101 plasmids, 2 µL of PRD1 (10^6^ pfu mL^−1^) and Ike (10^6^ pfu mL^−1^) phages were spotted on the solidified soft agar. The plates were incubated overnight at 37°C and examined for plaque formation indicative of phage sensitivity.

### Cohesion assay

Cells were grown overnight at 37°C in LB medium with appropriate antibiotics, subcultured by dilution (1:50) in fresh LB medium, and further incubated with shaking at 37°C for 6 h. Cultures were then removed from the incubator and kept undisturbed on the bench top for 3 h. The top 1 mL of the settled culture was removed for a spectrophotometric (OD_600_) reading. A cohesion index for each strain was calculated relative to that determined for the isogenic, plasmid-free strain MC4100 (set to a value of 1). Cohesion assays were repeated at least three times in triplicate; a representative experiment is shown, with replicate data points and average transfer frequencies as bars, along with standard deviations as error bars.

### Pilus labeling

Pili were labeled as described previously (11), with important exceptions noted below. Host cells carrying pOX38 variants and pKKF082 or pKM101 variants and pKKF090 were grown overnight in the presence of LB medium and appropriate antibiotics. Strains were subcultured by dilution (1:50) in antibiotic-free LB medium and incubated in static conditions at 37°C for 2 h. Sodium salicylate (1 mM final concentration) was added to induce expression of genes encoding the Cys-derivatized pilin proteins. Cultures were incubated without shaking for 3 h at 37°C, then AlexaFluor 488 C_5_ Maleimide (AF488-mal, Thermo Fisher Scientific) was added to 100 μL of cells at a final concentration of 25 μg mL^−1^. After 30 min of incubation at 37°C without shaking, the cells were washed once with prewarmed physiologically buffered saline (PBS) and resuspended in 20 µL PBS maintained at 37°C. Labeled cells (3 μL) were imaged with an Olympus IX81 microscope equipped with a UPLSAPO 100× objective lens (Oil Immersion), a Hamamatsu ORCA camera, and SlideBook 6 imaging software with FITC and DIC filters. In our prior study (11), which focused on monitoring phage-induced release of F pili from pOX38-carrying cells, we diluted overnight-grown strains carrying pOX38 or pOX38Δ*traD* by 1:1,000 and then subcultured for ~2 h with shaking. We then kept cells at room temperature and then ice for phage infections, monitoring pilus release at 5-min intervals for 20 min. To both phage-infected and uninfected controls, we then added GFP-labeled MS2 phage (MS2-GFP) for visualization of F pili. Although this protocol sufficed to show that the pOX38Δ*traD* mutant elaborates aberrantly long pili (11), it underestimated both the lengths and numbers of cell-associated F pili due to (i) extensive shaking during subculturing (causing breakage of pili and subsequent loss during pelleting of cells by centrifugation), (ii) maintenance of cells at room temperature and further chilling prior to MS2 infection and MS2-GFP labeling (conditions known to induce nonspecific release of F pili [76]), and (iii) addition of MS2-GFP for pilus labeling (MS2 binding triggers release of F pili from cells).

### Pilus quantification by ImageJ/microbeJ

Lengths of labeled pili and pilus fragments were measured from FITC images using ImageJ/Fiji software (version 1.54p). The images were converted to 8-bit format, background fluorescence was subtracted, noise reduction was carried out using the “Despeckle” filter, and the contrast was adjusted via CLAHE. The “filament” detection mode in MicrobeJ was used to detect individual pili/fragments, with lengths automatically measured. Fluorescent cells and misidentified pili or clusters of pili were manually corrected to increase the accuracy of the detection. Pilus/fragment length data were exported as CSV files for further analysis using GraphPad Prism software (version 10).

### Plotting frequency distribution histograms

The frequency distribution of pilus/fragment lengths measured from four FITC images of each strain using GraphPad Prism software (version 10) was presented in histogram format. A bin range of 0.5–14.5 with a width of 1.0 was used. The results were displayed as a bar graph, where the *y*-axis represents the number of fluorescent pili/fragments (fluorescent objects) and the *x*-axis represents pilus/fragment lengths in µm.

### Statistical analyses

Statistical analyses of pilus production (total number of fluorescent objects visualized in four representative fields) by strains carrying the WT F or pKM101 plasmids were compared with those elaborated by mutant strains with GraphPad Prism (version 10), using the nonparametric Kruskal–Wallis test followed by Dunn’s multiple-comparisons *post hoc* test. Significance is indicated as follows: not significant (ns), *P* > 0.05; *P < 0.05; **P < 0.01; ***P < 0.001;, ****P < 0.0001.

## Data Availability

The authors declare that all data supporting the findings of this study are available within the paper and its supplemental files.

## SUPPLEMENTAL MATERIAL

Supplemental Material mbio.01368-26-s0001.pdf File S1, Tables S1 and S2, and Figures S1 to S4.

ASM does not own the copyrights to Supplemental Material that may be linked to, or accessed through, an article. The authors have granted ASM a non-exclusive, world-wide license to publish the Supplemental Material files. Please contact the corresponding author directly for reuse.
