# Supplementary material for: VirD4 coupling proteins suppress accumulation of conjugative pili produced by type IV secretion systems
Source: mBio. 2026 Jul 16;17(8):e01368-26. doi: 10.1128/mbio.01368-26 (PMC13463995; doi:10.1128/mbio.01368-26)
Supplement: Supplementary file 1 [file mbio.01368-26-s0001.pdf]

Supplementary File S1. Plasmid Constructions.

E. coli strain DH5α carrying pKM101-Spc and pKD46 was used to generate the pKM101Δ*oriT*-Δ*traKJI::*FRT-*kan*^{R}-FRT variant by recombineering. A FRT-*kan*^{R}-FRT cassette from pKD13 was amplified with oligonucleotides with sequence homologies upstream and downstream of the region targeted for deletion. The λ *red-gam* system encoded by pKD46 was induced with 0.1% arabinose (final concentration), and the FRT-*kan*^{R}-FRT amplicon of interest was introduced into cells by electroporation. Resulting recombinants were selected on LB agar supplemented with kanamycin. Because pKM101 is a multicopy plasmid, plasmids lacking the integrated *kan*^{R} cassette were eliminated by subculturing the *kan*^{R} transformants for 4 days in LB medium supplemented with a high concentration (200 μg ml^{-1}) of kanamycin. Strains with the desired recombinant plasmid were identified by PCR amplification using oligonucleotides listed in Table S2 and further confirmed by Sanger sequencing across the deleted junction.

The pKM101, pOX38 and pED208 *tra* genes were amplified by PCR using plasmid templates and oligonucleotides listed in Tables S1 and S2. The amplicons were digested and cloned in pBAD24 or pKG116. Deletion and substitution mutations were made using the respective parent plasmid as template and oligonucleotides listed in Table S2. pKKF089 is a pKG116-based plasmid expressing $traM_{pKM}$ from P*nahG*. $traM_{pKM}$ along with upstream and downstream sequences was amplified, the amplicon was digested with NdeI and BamHI, and the digested fragment was introduced into similarly-digested pKG116. pKKF090 expressing P*nahG*::$traM_{pKM}S91C$ was generated from pKKF089 by inverse PCR. pYGL205 expressing P_{BAD}::$traJ_{pKM}\Delta AAD$ was generated from pYGL22 by inverse PCR. pKKF081 expressing P*nahG*::*traY'-traA-traL'* was amplified from pOX38, the amplicon was digested with NdeI and BamHI , and the digested fragment was introduced into similarly-digested pKG116. pKKF082 expressing P*nahG*::*traY'-*$traA_FA113C$*-traL'* was generated from pKKF081 by inverse PCR. pYGL340 expressing P_{BAD}::$traD_FK198Q$ was generated from pPK31 by inverse PCR. For construction of pAM143 expressing P_{BAD}::$traD_F\Delta CT15$, $traD_F$ lacking 45 nucleotides at the 3' end was amplified from pOX38, the amplicon was digested with KpnI and HindIII, and the digested fragment was introduced into similarly-digested pBAD24. pPK100 expressing P_{BAD}::$traJ_{pKM}\Delta GKT$ (residues 138-140) was constructed using Gibson assembly (NEB). A fragment lacking the region to be deleted (G138, K139 and T140) was amplified using pYGL22 as template. A shorter fragment (obtained by annealing oligonucleotides traJ_delGKT_F and traJ_delGKT_R) was ligated to this longer fragment using Gibson assembly. pPK101 expressing P_{BAD}::$traD_F\Delta CTD$ was constructed by amplification of $traD_F\Delta CTD$ from pYGL492, the amplicon was digested with NheI and HindIII, and the digested fragment was introduced into similarly-digested pBAD24. pYGL762 is a spectinomycin-resistant (*spc*^{R}) cloning vector carrying IPTG inducible P_{tac} promoter. It was constructed by PCR amplification of the inducible promoter sequence using pRR48 as a template, and PCR amplification of *spc*^{R} along with some pSC101 sequence using pGB2 as a template. Both fragments were ligated by Gibson assembly. pYGL764 is a gentamicin-resistant (*gen*^{R}) version of pYGL762. It was constructed by ligating PCR-amplified pYGL762 lacking *spec*^{R} and the PCR-amplified *gen*^{R} fragment obtained from pYGL680 by Gibson assembly. pYGL246 expressing P_{BAD}::$traD_FCTD_{ED}$ was constructed by PCR amplification of CTD_{ED} (nucleotide sequence encoding the last 166 amino acids of $TraD_{pED}$) using pPK18 as a template, PCR amplification of pPK31 lacking CTD_F (nucleotide sequence lacking the last 148 amino acids of $TraD_F$), and ligation of the KpnI- and HindIII-digested PCR fragments. pYGL247 expressing P_{BAD}::$traD_{ED}CTD_F$ was constructed by PCR amplification of CTD_F using pPK31 as a template, PCR amplification of pPK18 lacking CTD_{ED}, and ligation of the KpnI- and HindIII-digested PCR fragments. pYGL843 expressing P_{tac}::$traD_F\Delta NTD$ was constructed by PCR amplification of *traD*Δ*NTD* using pPK31 as a template, PCR amplification of the cloning vector using pYGL764 as a template, and ligation of the PCR fragments by Gibson assembly.

Table S1. Strains and plasmids used in this study.

Strains or plasmids	Relevant characteristics	Source
**Strains**		
DH5α	F^- *endA1 glnV44 thi-1 recA1 relA1 gyrA96 deoR nupG purB20* φ80*dlacZ*ΔM15 Δ(*lacZYA-argF*)U169, hsdR17(r_K^- m_K^+), λ^-	Lab stock
MG1655	F^- $\lambda - ilvG - rfb - 50 rph - 1$	Lab stock
MC4100	F^- *[araD139]*$_{B/r}$ Δ*(argF-lac)169* λ^- *e14*$^-$ *flhD5301* Δ*(fruK-yeiR)725(fruA25) relA1 rpsL150(strR) rbsR22* Δ*(fimB-fimE)632(::IS1) deoC*	Lab stock
MC4100-*rif*^{R}	*rif*^{R} strain used as recipient in conjugation assays	Lab stock
**Plasmid vectors**		
pBAD24	*crb*^{R}, P_{BAD} cloning vector	[1]
pKG116	pACYC184 derivative with *nahG* promoter and RBS	[2]
pKD13	*crb*^{R}, R6K plasmid with FRT-*kan*^{R}-FRT	[3]
pKD46	*crb*^{R}, λ-Red recombinase expression plasmid	[3]
pRR48	*crb*^{R}, pBR322 derivative with Ptac promoter	[4]
pGB2	*spc*^{R}, pSC101 derivative	[5]
pYGL680	*gen*^{R}, ColE1 with Ptac promoter	[6]
pYGL762	*spc*^{R}, Ptac promoter on pGB2	This study
pYGL764	*gen*^{R}, Ptac promoter on pGB2	This study
**pKM101 variants**		
pKM101-Spc	*spc*^{R}, pKM101 derivative	[7]
pKM101Δ*traM*	*spc*^{R}, pKM101 deleted of *traM*	[8]
pKM101Δ*traB*	*spc*^{R}, pKM101 deleted of *traB*	[8]
pKM101Δ*traG*	*spc*^{R} *kan*^{R}, pKM101 deleted of *traG*	[8]
pKM101Δ*traJ*	*spc*^{R}, pKM101 deleted of *traJ*	[7]
pKM101Δ*oriT*-Δ*traKJI::*FRT-*kan*^{R}-FRT	*spc*^{R}*kan*^{R}, pKM101 deleted of *oriT-traKJI*	This study
**pOX38 variants**		
pOX38-Tc	*tet*^{R}, F deleted of HindIII fragment	[9]
pOX38Δ*traA*	*tet*^{R}, pOX38 deleted of *traA*	[10]
pOX38Δ*traD*	*tet*^{R}, pOX38 deleted of *traD*	[11]
pOX38-*traMK3*	*kan*^{R}, pOX38 with *kan*^{R} cassette inserted in SalI site of *traM*	[12]
**pED208 variants**		
pED208Δ*traA*	*spc*^{R}, pED208 deleted of *traA*	[13]
***tra* gene expression plasmids (pKM101)**		
pPK100	*crb*^{R}, pBAD24 expressing *traJ*$_{pKM}$Δ*GKT* (residues 138-140)	This study
pKKF089	*chl*^{R}, pKG116 expressing *traM*$_{pKM}$	This study
pKKF090	*chl*^{R}, pKG116 expressing *traM.S91C*$_{pKM}$	This study
pYGL22	*crb*^{R}, pBAD24 expressing *traJ*$_{pKM}$	[14]
pYGL33	*crb*^{R}, pBAD24 expressing *traJ-traI-traK*$_{pKM}$	[14]
pYGL50	*crb*^{R}, pBAD24 expressing *traK-traJ* $_{pKM}$	[14]
pYGL51	*crb*^{R}, pBAD24 expressing *traJ-traI* $_{pKM}$	[14]
pYGL205	*crb*^{R}, pBAD24 expressing *traJ* Δ*AAD*$_{pKM}$	This study
***tra* gene expression plasmids (pOX38)**		
pPK31	*crb*^{R}, pBAD24 expressing *traD*$_F$	[11]
pPK101	*crb*^{R}, pBAD24 expressing *traD*Δ*CTD*$_F$	This study
pKKF081	*chl*^{R}, pKG116 expressing *traY'-traA-traL'*$_F$	This study
pKKF082	*chl*^{R}, pKG116 expressing *traY'-traA.A113C-traL'*$_F$	This study
pYGL246	*crb*^{R}, pBAD24 expressing *traD*$_F$*CTD*$_{ED}$	This study
pYGL247	*crb*^{R}, pBAD24 expressing *traD*$_{ED}$*CTD*$_F$	This study

pYGL340	*crb*^{R}, pBAD24 expressing *traD.K198Q*$_F$	This study
pYGL492	*chl*^{R}, pKG116 expressing $_{strep}$*traD*Δ*CTD*$_F$	[15]
pYGL843	*gen*^{R}, pYGL764 expressing *traD*Δ*NTD*$_F$	This study
pAM143	*crb*^{R}, pBAD24 expressing *traD*Δ*CT15*$_F$	This study
***tra* gene expression plasmids (pED208)**		
pPK18	*crb*^{R}, pBAD24 expressing *traD*$_{ED}$	[13]

References:

1. Guzman, L.M., Belin, D., Carson, M.J. and Beckwith, J.O.N., 1995. Tight regulation, modulation, and high-level expression by vectors containing the arabinose PBAD promoter. *J. Bacteriol.*, *177*(14), pp.4121-4130.

2. Haeusser, D.P., Rowlett, V.W. and Margolin, W., 2015. A mutation in *Escherichia coli* fts Z bypasses the requirement for the essential division gene zip A and confers resistance to FtsZ assembly inhibitors by stabilizing protofilament bundling. *Mol. Microbiol.*, *97*(5), pp.988-1005.

3. Datsenko, K.A. and Wanner, B.L., 2000. One-step inactivation of chromosomal genes in *Escherichia coli* K-12 using PCR products. *Proc. Natl. Acad. Sci. USA*, *97*(12), pp.6640-6645.

4. Haeusser, D.P., Hoashi, M., Weaver, A., Brown, N., Pan, J., Sawitzke, J.A., Thomason, L.C., Court, D.L. and Margolin, W., 2014. The Kil peptide of bacteriophage λ blocks *Escherichia coli* cytokinesis via ZipA-dependent inhibition of FtsZ assembly. *PLoS Genetics*, *10*(3), p.e1004217.

5. Churchward, G., Belin, D. and Nagamine, Y., 1984. A pSC101-derived plasmid which shows no sequence homology to other commonly used cloning vectors. *Gene*, *31*(1-3), pp.165-171.

6. Li, Y.G., Haeusser, D., Margolin, W. and Christie, P.J., 2025. Conjugative delivery of toxin genes *ccdB* and *kil* confers synergistic killing of bacterial recipients. *J. Bacteriol.*, pp.e00168-25.

7. Whitaker, N., Berry, T.M., Rosenthal, N., Gordon, J.E., Gonzalez-Rivera, C., Sheehan, K.B., Truchan, H.K., VieBrock, L., Newton, I.L., Carlyon, J.A. and Christie, P.J., 2016. Chimeric coupling proteins mediate transfer of heterologous type IV effectors through the *Escherichia coli* pKM101-encoded conjugation machine. *J. Bacteriol.*, *198*(19), pp.2701-2718.

8. Gordon, J.E., Costa, T.R., Patel, R.S., Gonzalez-Rivera, C., Sarkar, M.K., Orlova, E.V., Waksman, G. and Christie, P.J., 2017. Use of chimeric type IV secretion systems to define contributions of outer membrane subassemblies for contact-dependent translocation. *Mol. Microbiol.*, *105*(2), pp.273-293.

9. Anthony, K.G., Sherburne, C., Sherburne, R. and Frost, L.S., 1994. The role of the pilus in recipient cell recognition during bacterial conjugation mediated by F-like plasmids. *Mol. Microbiol.*, *13*(6), pp.939-953.

10. Kishida K, Bosserman RE, Harb L, Khara P, Song L, Hu B, et al. Contributions of F-specific subunits to the F plasmid-encoded type IV secretion system and F pilus. *Mol Microbiol.* 2022;117(5):1275-90.

11. Harb, L., Chamakura, K., Khara, P., Christie, P.J., Young, R. and Zeng, L., 2020. ssRNA phage penetration triggers detachment of the F-pilus. *Proc. Natl. Acad. Sci. USA*, *117*(41), pp.25751-25758.

12. Penfold, S.S., Simon, J. and Frost, L.S., 1996. Regulation of the expression of the *traM* gene of the F sex factor of *Escherichia coli. Mol. Microbiol.*, *20*(3), pp.549-558.

13. Hu, B., Khara, P. and Christie, P.J., 2019. Structural bases for F plasmid conjugation and F pilus biogenesis in *Escherichia coli. Proc. Natl. Acad. Sci. USA*, *116*(28), pp.14222-14227.

14. Li, Y.G. and Christie, P.J., 2020. The TraK accessory factor activates substrate transfer through the pKM101 type IV secretion system independently of its role in relaxosome assembly. *Mol. Microbiol.*, *114*(2), pp.214-229.

15. Kishida, K., Li, Y.G., Ogawa-Kishida, N., Khara, P., Al Mamun, A.A.M., Bosserman, R.E. and Christie, P.J., 2024. Chimeric systems composed of swapped Tra subunits between distantly-related F plasmids reveal striking plasticity among type IV secretion machines. *PLoS Genetics*, *20*(3), p.e1011088.

Table S2. Oligonucleotides used in this study

Primer name	Sequence (5' to 3')	Purpose (plasmid constructions, screening and confirmations)
Ptac_F:	AAATCTAGCGAGGGCTTTACCAGTATACACTCCGCTATCGCTACGTGAC	pYGL762 construction
Ptac_R:	GCTTACCCGTCTTACTGTCGGCCACCTGACGTCTAAGAAACCATTATTATCATGAC	pYGL762 construction
pGB2_F:	CGACAGTAAGACGGGTAAGCCTGTTG	pYGL762 construction
pGB2_R:	GTAAAGCCCTCGCTAGATTTTAATGCGGATG	pYGL762 construction
gent-V_F:	GTCGCTGCCGACTGGGCAATGGAG	pYGL764 construction
gent-V_R:	GCGCCGTTACCACCGCTGCGTTC	pYGL764 construction
gent-I_F:	CGCAGCGGTGGTAACGGCGCCATGATAATAATGGTTTCTTAGACGTCAGGTGGCAC	pYGL764 construction
gent-I_R:	ATTGCCCAGTCGGCAGCGACGGTCTGACAGTTAGGTGGCGGTACTTGG	pYGL764 construction
oriTKJI_FRT_Km_F	GCCTCAAGCCCAAAGAAACGGTCAATTTCTGCGGCCGTGGTTTTCGGGCTATTCCGGGGATCCGTCGACC	pKM101Δ*oriT*-Δ*traKJI*::FRT-*kan*^{R}-FRT construction
oriTKJI_FRT_Km_R	AACCTACGTAGATCGCTCCTACCAAAAAAGATGATTTTTTATCCCTTAACTGTAGGCTGGAGCTGCTTCG	pKM101Δ*oriT*-Δ*traKJI*::FRT-*kan*^{R}-FRT construction
mid_traI_F	GATCGGGTTTCTAAACTGGTATCGCGGC	pKM101Δ*oriT*-Δ*traKJI*::FRT-*kan*^{R}-FRT screening and confirmation
mid_oriT_R	CATGCAAGCATGAATACGGAATATTGAACGC	pKM101Δ*oriT*-Δ*traKJI*::FRT-*kan*^{R}-FRT screening and confirmation
Seq_FRT-Km_F	CTTCCTCGTGCTTTACGGTATCGCC	pKM101Δ*oriT*-Δ*traKJI*::FRT-*kan*^{R}-FRT confirmation
Seq_FRT-Km_R	CTGACATTCATCCGGGGTCAGCAC	pKM101Δ*oriT*-Δ*traKJI*::FRT-*kan*^{R}-FRT confirmation
traJ_delGKT_F	GCCGGTACAACCGGTACTACAATTTTCAATGAACTG	pPK100 construction
traJ_delGKT_R	CAGTTCATTGAAAATTGTAGTACCGGTTGTACCGGC	pPK100 construction
itraJ_F	ACAATTTTCAATGAACTGTTATTTAAGAGCATC	pPK100 construction
itraJ_R	AGTACCGGTTGTACCGGCAATCGAAAAATG	pPK100 construction
KKTX296	CGCAAGCTTCATATGTGATTTAAGGCTACTGGCAGAG	pKKF089 construction
KKTX297	CGCGGATCCAGCGCGCGAGGTAAGCGAGTCGC	pKKF089construction
KKTX164	TGCCTGACGGGCGTAGGAAGCTG	pKKF090 construction
KKTX165	AACCAGATATGATGCAGAACC	pKKF090 construction
TraJDAAD_F	AATTTTTCCCTGCGTGACTGGCTTGATGAT	pYGL205 construction
TraJDAAD_R	TTCAGTGCGTTTATCATAGGCGTTTAAAATAACATC	pYGL205 construction
traD_CTD_NheI_F	CTAGCTAGCAGGAGGAATTCACCATGTGGTCTCATCCTCAATTCGAAAAGGGTACCAG	pPK101 construction
traD_CTD_HindIII_R	CCCAAGCTTTCAAGCGACCTTCGGTCGTGTCTGATATTTC	pPK101construction
KKTX287	CGCGGATCCCTATTTAATCCACTGCCGGAAACACG	pKKF081 construction
KKTX288	CGCAAGCTTCATATGTTGAAAAGATTTGGTACACGTTCTGCAACAG	pKKF081 construction
KKTX285	AATAAATACAGAGATGATGGCAAAACCGG	pKKF082 construction
KKTX286	TGTGTGGGTATGGCCGTCGTTGGCCTCTG	pKKF082 construction
TraD-OX_CT166-ED_inst_KpnI_F	ATATATGGTACCGACGCCCTGCTGTTGCGGGATG	pYGL246 construction

TraD-OX_CT166-ED_inst_HindIII_R	ACAGCCAAGCTTCATCAGTATTCCCTCC	pYGL246 construction
TraD-OX_CT166-ED_vctr_HindIII_F	TTCTGAAAGCTTGGCTGTTTTGGCGGATG	pYGL246 construction
TraD-OX_CT166-ED_vctr_KpnI_R	ATATATGGTACCAGCGACCTTCGGTCGTGTCTGATATTTC	pYGL246 construction
TraD-ED_CT148-OX_inst_KpnI_F	ATATATGGTACCCCGGAGTTTATTCCGCGTGACATCAAC	pYGL247 construction
TraD-ED_CT148-OX_inst_HindIII_R	ACAGCCAAGCTTTCAGAAATCATCTCCCG	pYGL247 construction
TraD-ED_CT148-OX_vctr_HindIII_F	CTGATGAAGCTTGGCTGTTTTGGCGG	pYGL247 construction
TraD-ED_CT148-OX_vctr_KpnI_R	ATATATGGTACCAGCCACCTTAGGCATCGAGTCATACTTC	pYGL247 construction
TraD-OX_K198Q_F	CAGTCGGAAGTTATCCGTCGTCTGG	pYGL340 construction
TraD-OX_K198Q_R	ACCGGCCCCGACCGTGC	pYGL340 construction
V764_F	GCTCCTCGAGGATCCGCGGATAAATAAG	pYGL843 construction
V764_R:	GCTGCAGATATCATGCATATGACACTCCTTTAAATTGTG	pYGL843 construction
TraD-DTM_F:	ATATGCATGATATCTGCAGCATGAATGAAGTCACAGGTGGTCGTCAGCTGAC	pYGL843 construction
TraD_R:	TCCGCGGATCCTCGAGGAGCTCAGAAATCATCTCCCGGCTCAACATCCTC	pYGL843 construction
AA394_traD_delC15OX_KpF	AAACTAGGTACCAGGAGGAATTCACCATGAGTTTTAACGCAAAGGATATGACCC	pAM143 construction
AA395_traD-dlC15OX_HdR	AACCTAAAGCTTTCAATTAATGTTCACCTCTTCACGACGC	pAM143 construction

F-encoded TraA pilin

MNAVLSVQGASAPVKKKSFFSKFTRLNMLRLARAVIPAAVLMMFFPQLAMA_AGSSGQ
DLMASGNTTVKATFGKDSSVVKWVVLAEVLVGAVMYMMTKNVKFLAGFAIISVFI**A**VG
MAVVGL

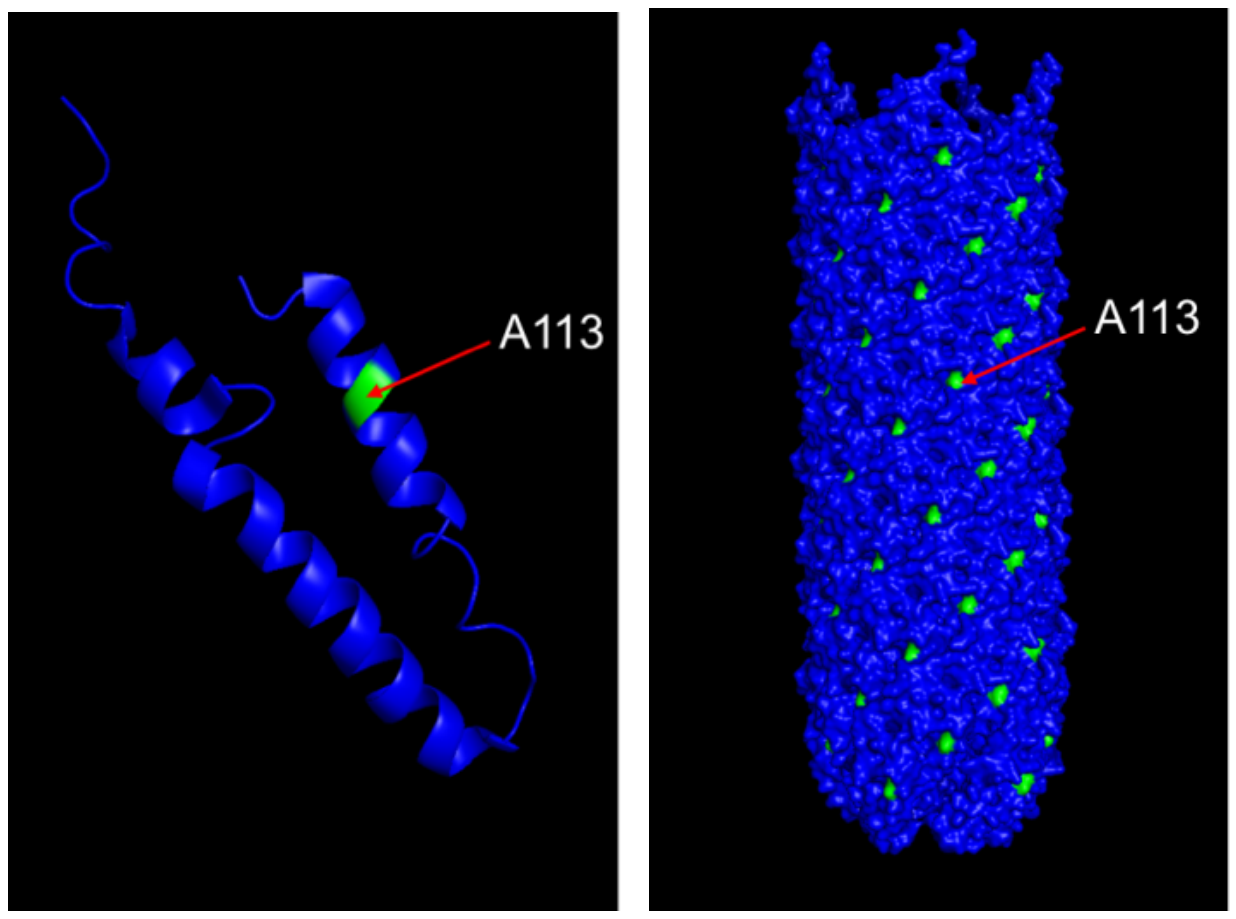

pKM101-encoded TraM pilin

MTTLFKKYGPAVVMGVLSIALPQIALA_AGTDTGESTATSIQTWLSTWIPIGCAIAIMVSCFM
WMLHVIPASFIPRIVISLIGIGSASYLV**S**LTGVGS

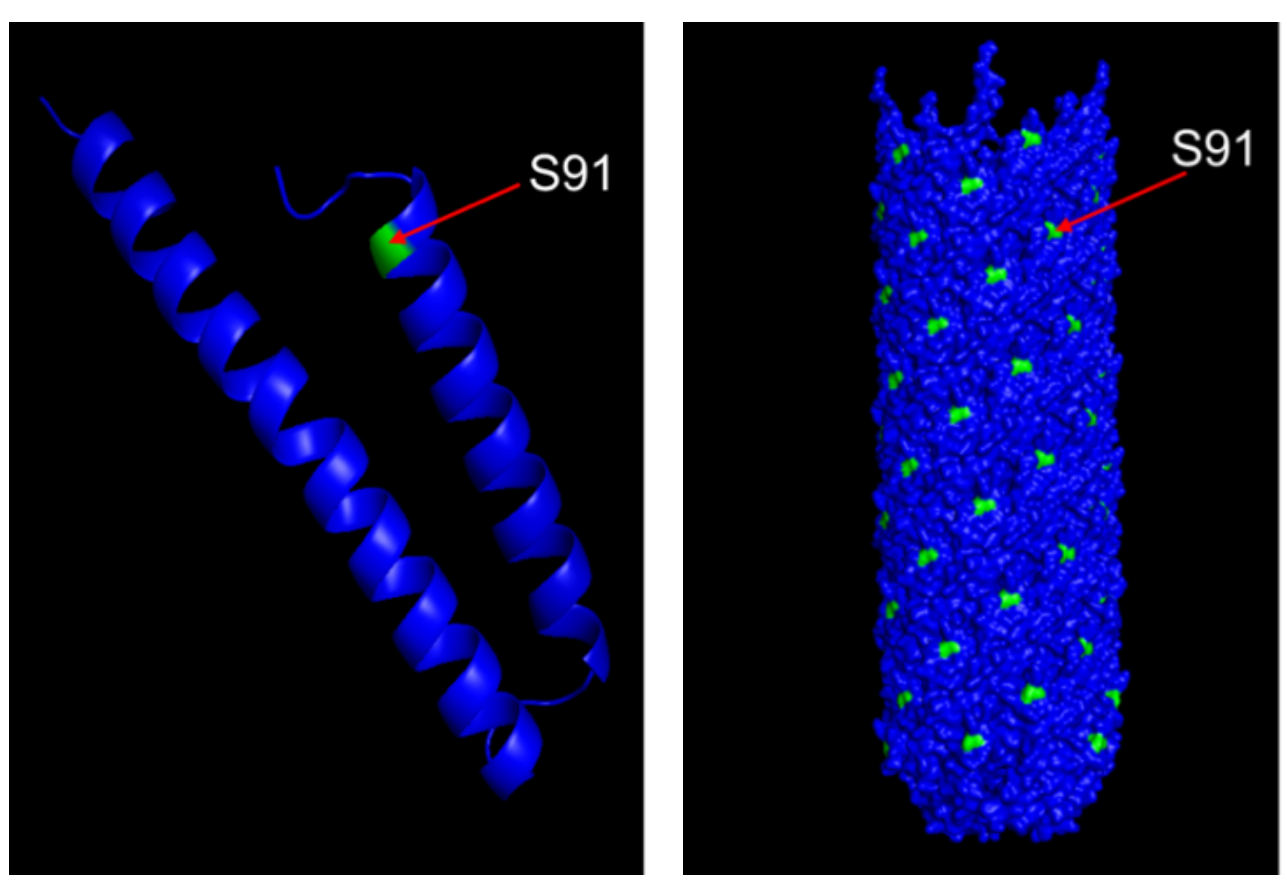

Fig. S1. Positions of Cys-substituted residues in the TraA and TraM pilins. TraA and TraM sequences are shown. Dash marks the signal sequence cleavage sites. Red residues denote positions of Cys substitutions. The TraA.A113C and TraM.S91C substitutions at the positions shown (bold red, green highlight) exhibited WT function for plasmid transfer, pilus production, and infection by the male-specific bacteriophages. Positions of these residues in the pilin monomers and within the assembled F and N pili are shown (see Costa, T.R.D., et al., 2016. *Cell* 166:1436-1444 & Amro, J., et al., 2023. *Structure* 31:375-384).

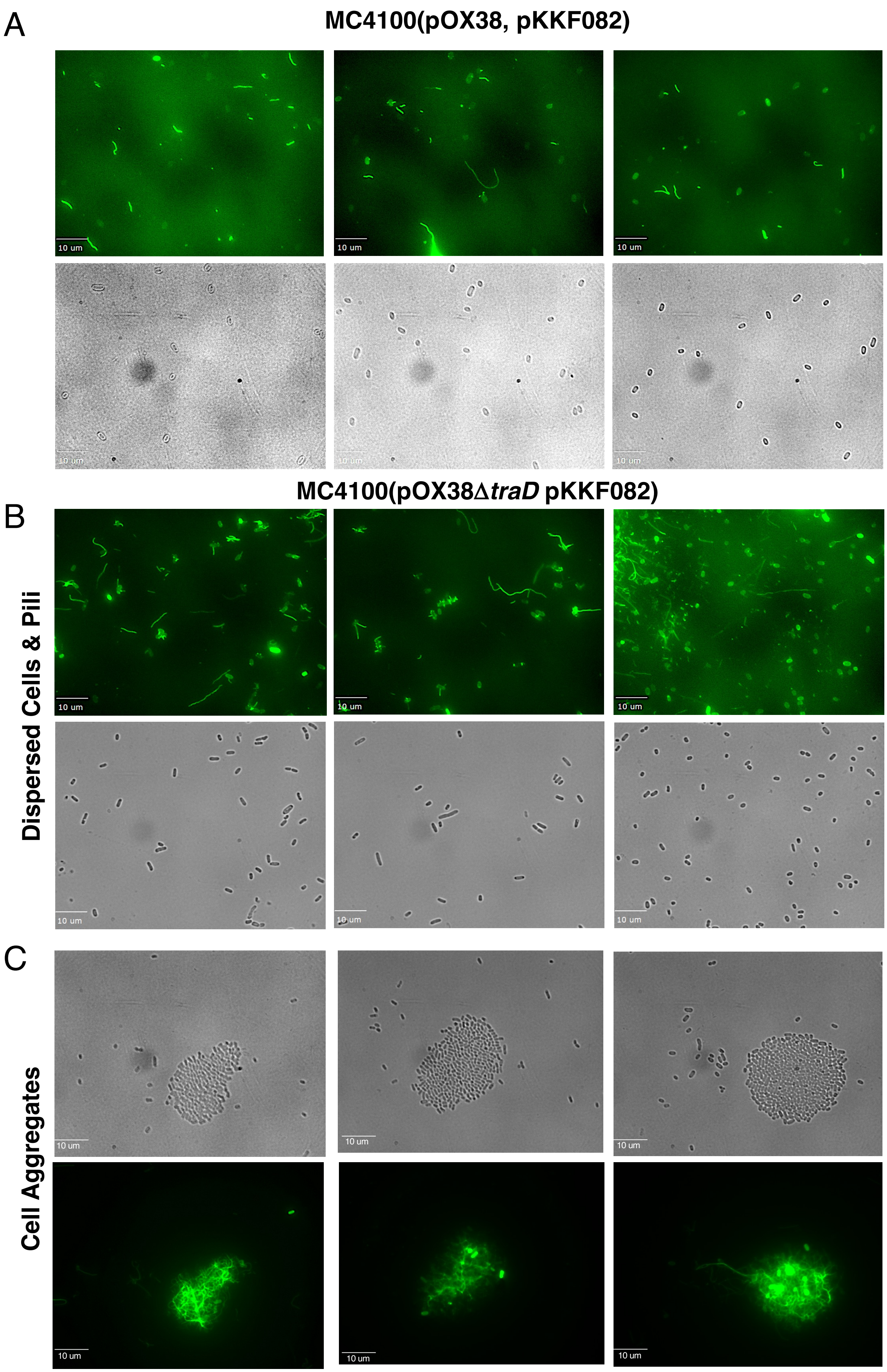

Fig. S2. Representative fields of view for strains indicated.

MC4100(pOX38Δ*traD* pKKF082)

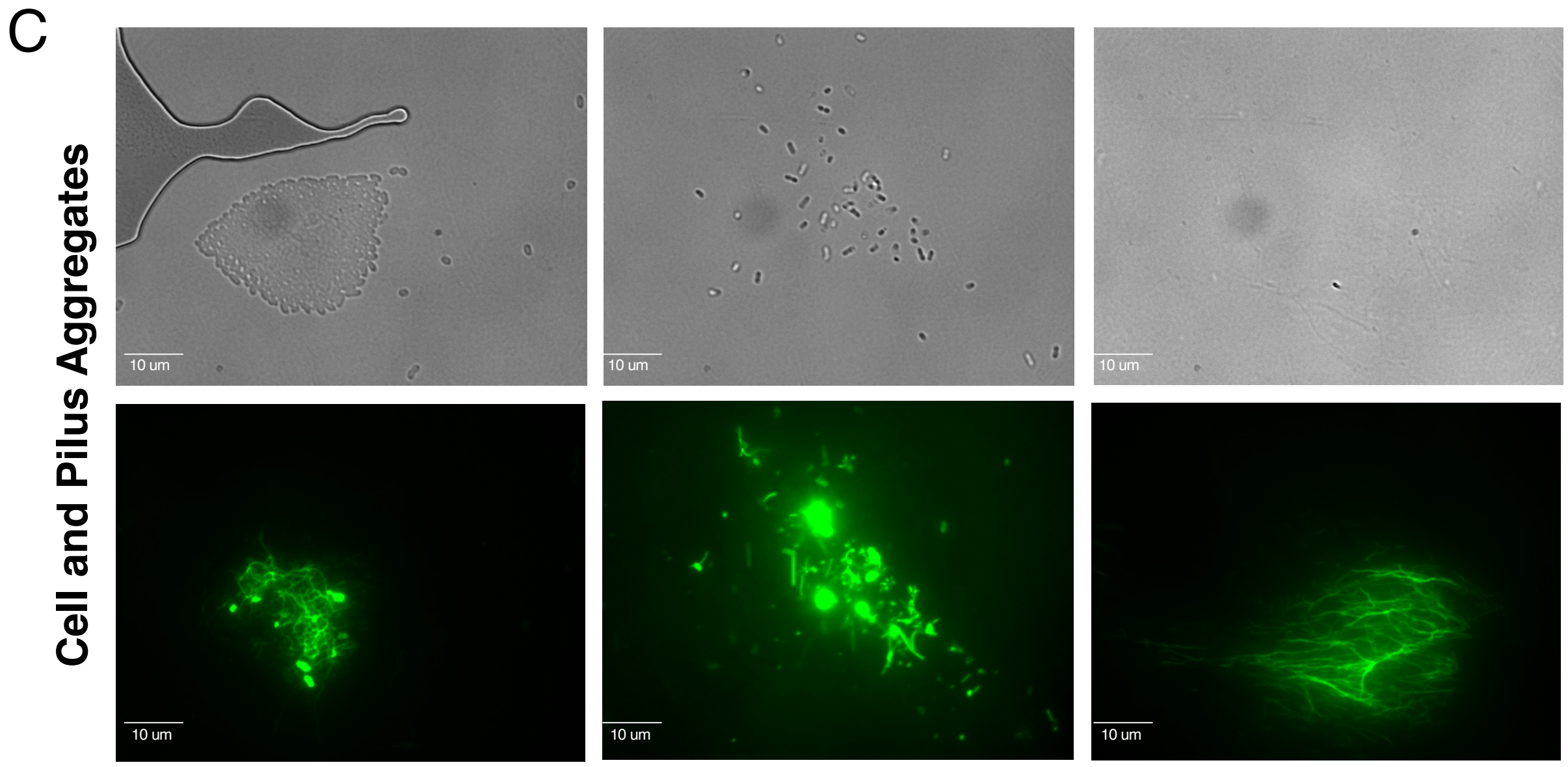

MC4100(pOX38Δ*traD*, pPK31, pKKF082)

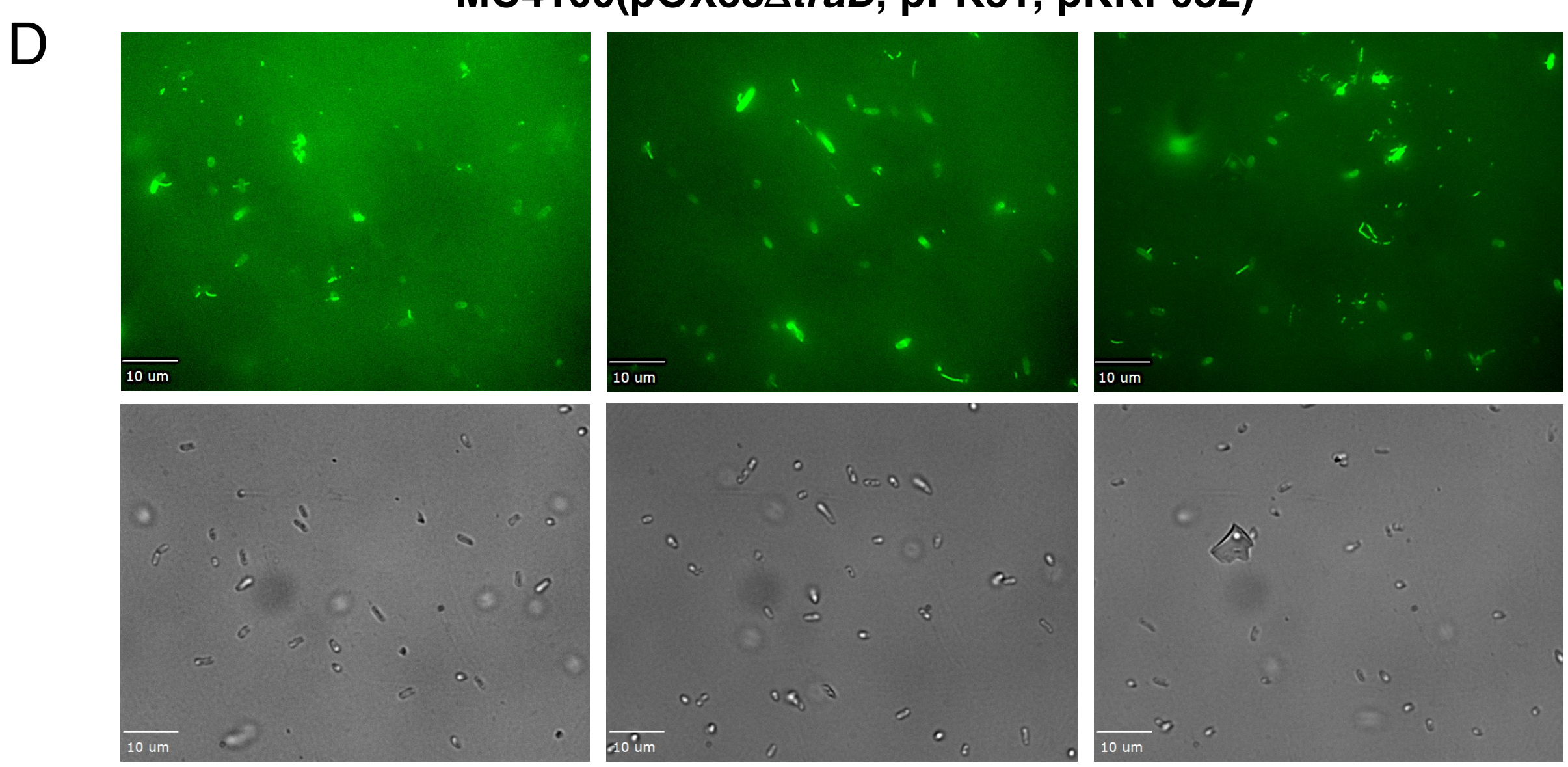

Fig. S2. Representative fields of view for strains indicated.

H

DH5α(pKKF090)

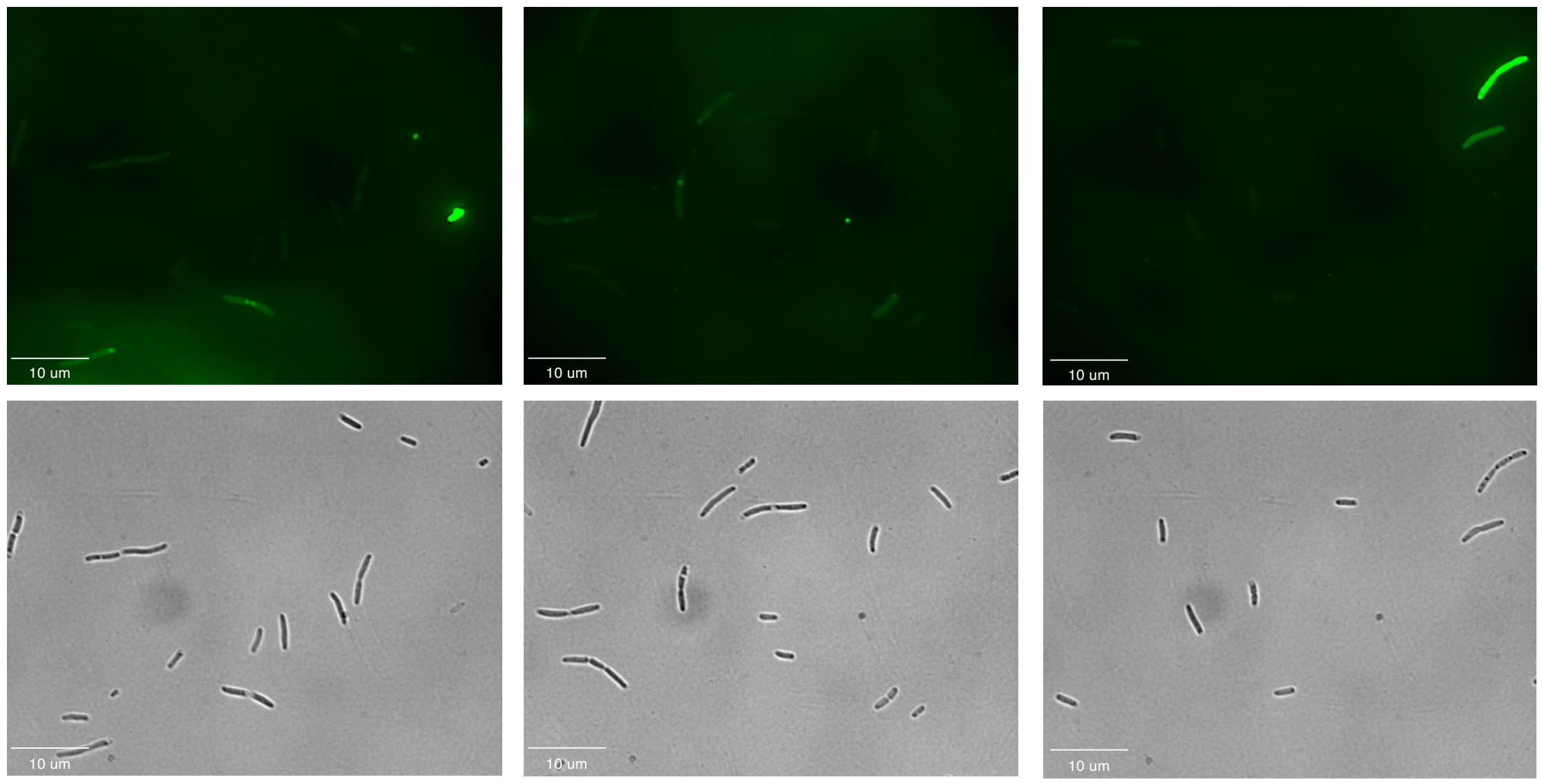

I

MG1655(pKM101Δ*traB*, pKKF090)

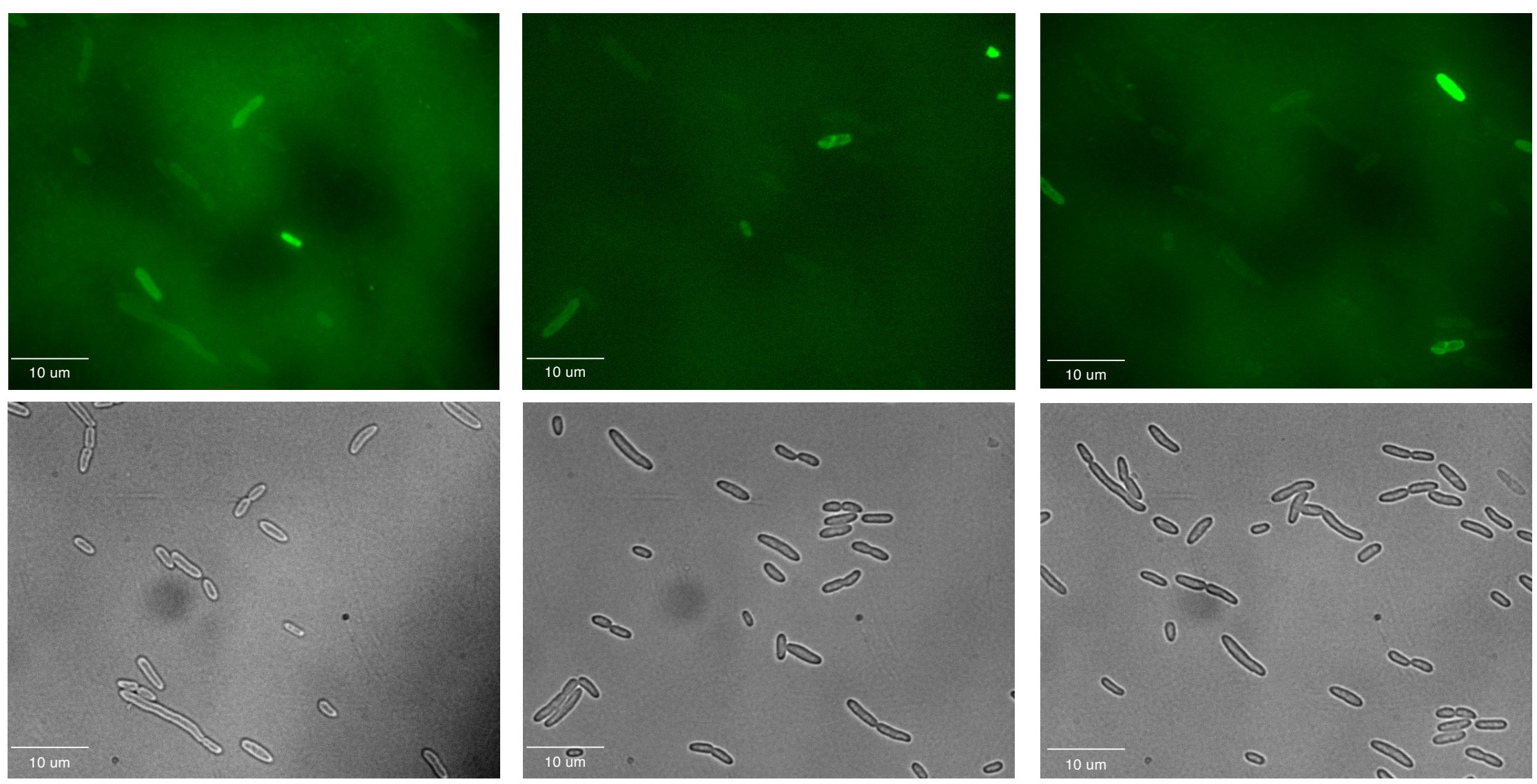

J

MG1655(pKM101Δ*traG*, pKKF090)

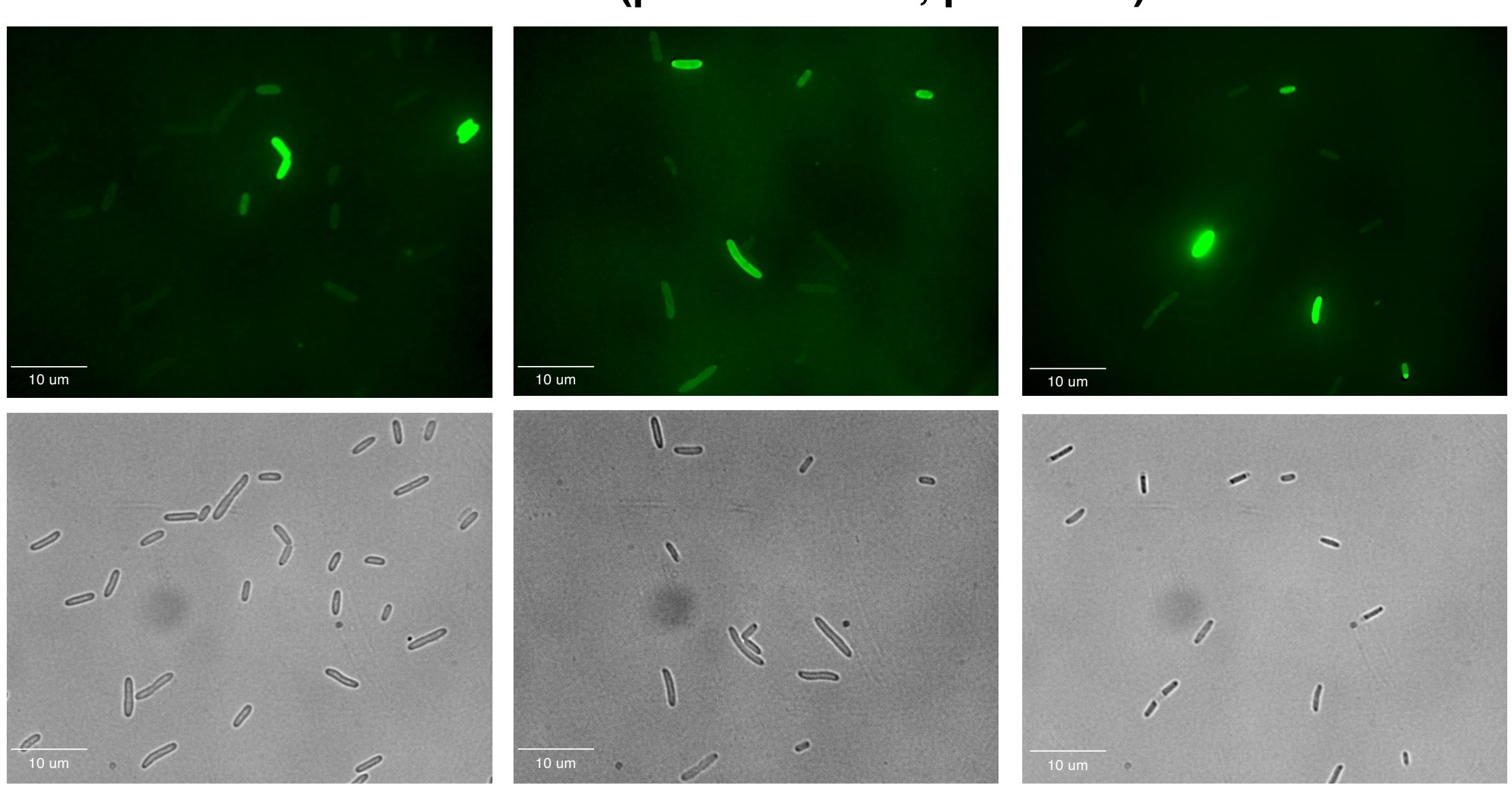

Fig. S2. Representative fields of view for strains indicated.

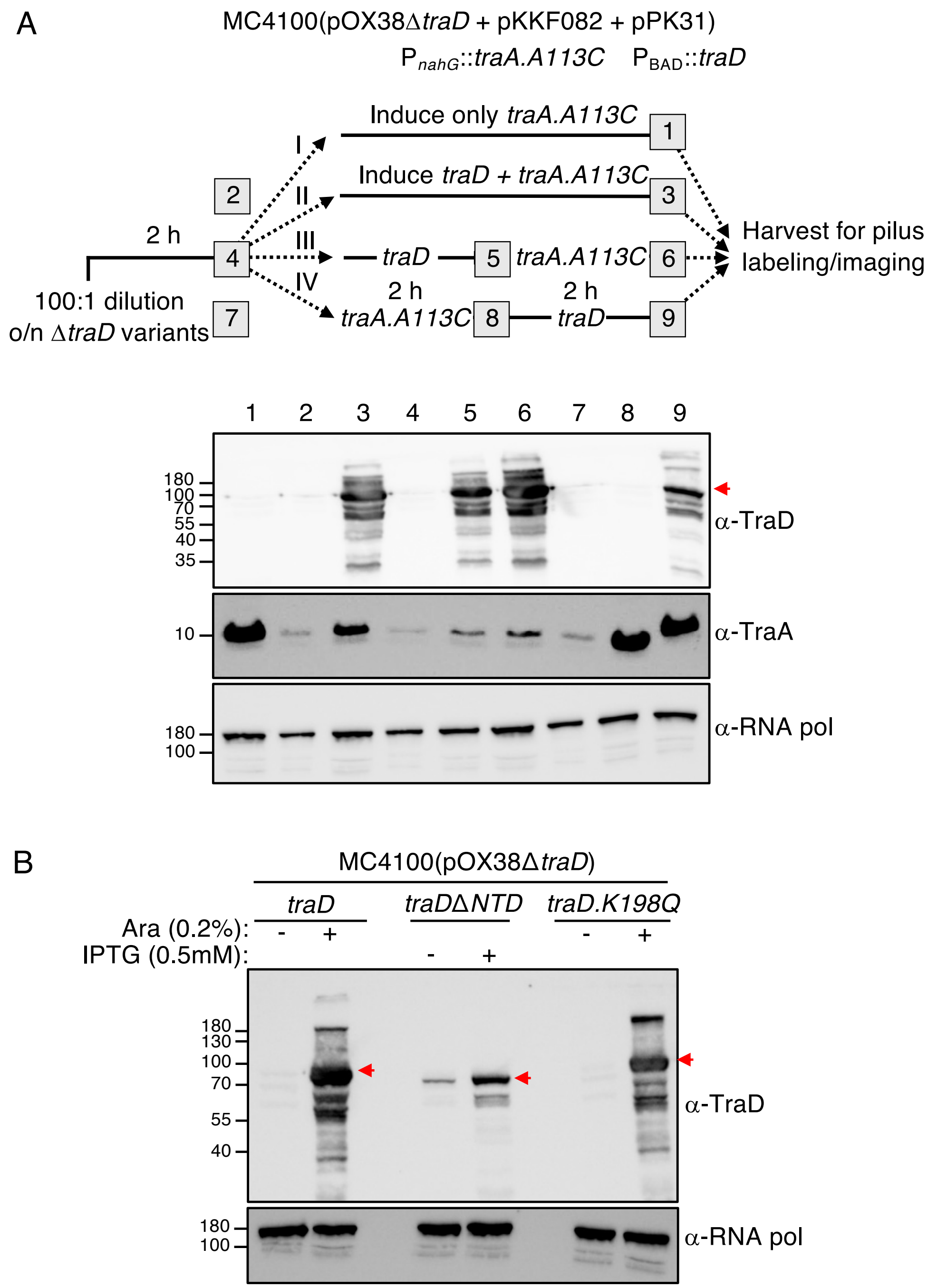

Fig. S3. A. Abundance of TraD and TraA in the pOX38Δ*traD* mutant listed at the top. Cells were grown and induced for expression of *traA.S11C* and/or *traD* according to the schematic (upper). At the time points indicated by numbers with gray outlines, cells were extracted and whole cell lysates were analyzed by immunoblotting (lower) for production of TraD and TraA. Note: The Δ*traD* mutant produces native TraA at low levels compared to inducibly produced TraA.S113C. **B.** Abundance of TraD mutant proteins produced in MC4100(pOX38Δ*traD*) carrying plasmids for expression of *traD* (pPK31), *traD*Δ*NTD* (pYGL843), or *traD.K198Q* (pYGL340). Strains were analyzed for production of the TraD variants shown without (-) and with (+) induction with arabinose or IPTG. **A & B.** Red arrows denote positions of TraD variants. RNA polymerase β subunit (RNAP) served as a loading control. MW markers (in kDa) at left.

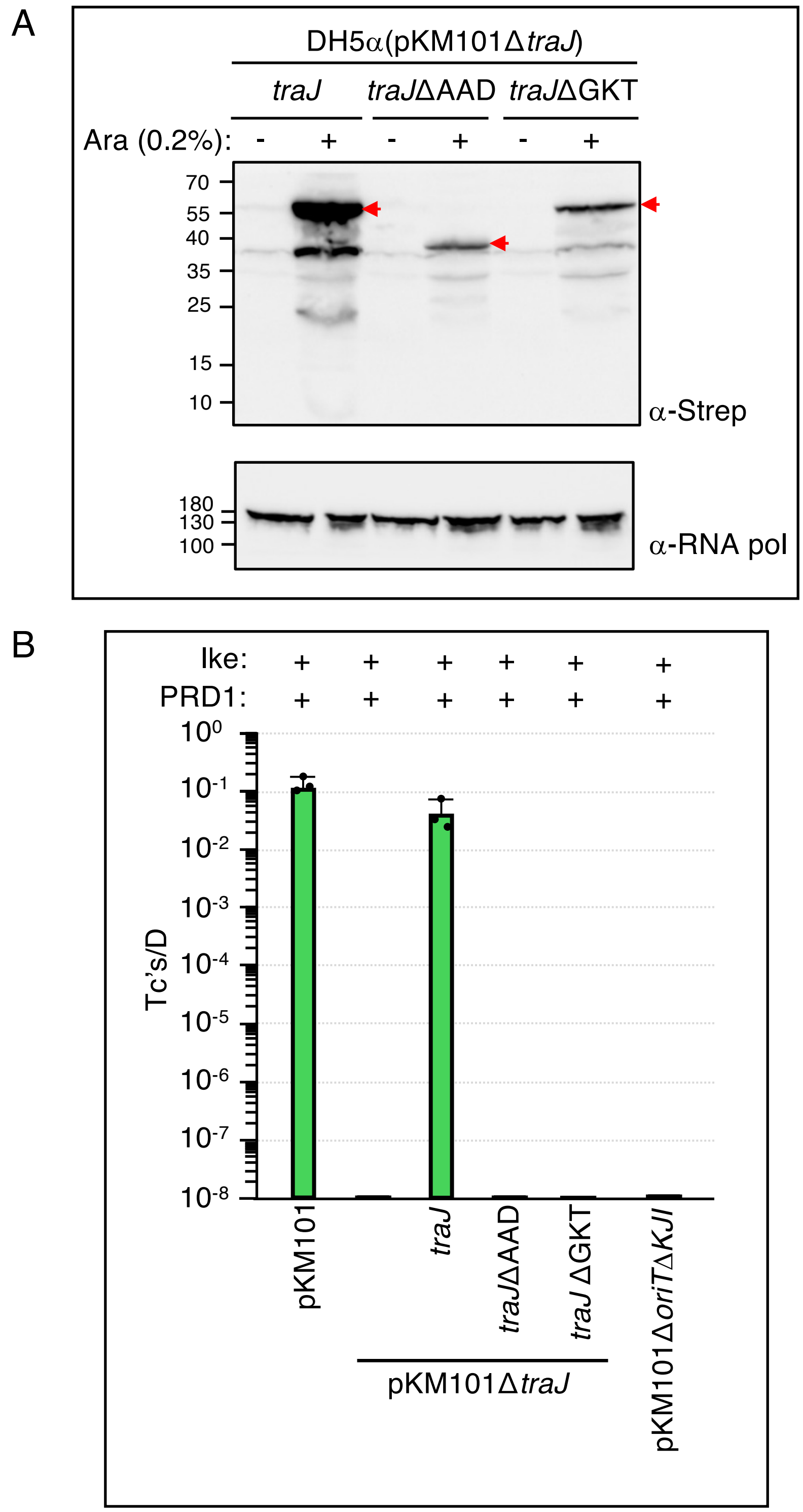

Fig. S4. Abundance and phenotypes of TraJ mutant proteins. **A.** Immunoblots showing production of Strep-tagged forms of TraJ, TraJΔAAD, and TraJΔGKT (highlighted by red arrows) upon arabinose induction of the corresponding genes from the P_{BAD} promoter. MW markers (in kDa) at left. RNA polymerase β subunit (RNAP) served as a loading control. **B.** Transfer frequencies and Ike and PRD1 phage sensitivity profiles of strains carrying the pKM101 variants shown, and expressing the *traJ* alleles from separate plasmids as indicated. Tc's/D, transconjugants per donor, resulting from 1 h matings in liquid. Matings were repeated at least three times in triplicate; a representative experiment is shown with replicate data points and the average transfer frequencies as bars along with standard deviations as error bars.
